# DNA methylation profiles of long-term cannabis users in midlife: a comprehensive evaluation of published cannabis-associated methylation markers in a representative cohort

**DOI:** 10.1038/s41380-025-03042-9

**Published:** 2025-06-27

**Authors:** Madeline H. Meier, Karen Sugden, Terrie E. Moffitt, Benjamin S. Williams, Kyle J. Bourassa, Renate Houts, Sandhya Ramrakha, Reremoana Theodore, Avshalom Caspi

**Affiliations:** 1 Department of Psychology, Arizona State University, Tempe, AZ USA; 2 Department of Psychology & Neuroscience, Duke University, Durham, NC USA; 3 Department of Psychiatry and Behavioral Sciences, Duke University School of Medicine, Durham, NC USA; 4 Center for Genomic and Computational Biology, Duke University, Durham, NC USA; 5 Social, Genetic and Developmental Psychiatry Centre, Institute of Psychiatry, Psychology and Neuroscience, King’s College London, London, England; 6 PROMENTA Research Center, University of Oslo, Oslo, Norway; 7 Mid-Atlantic Mental Illness Research, Education and Clinical Center, Durham VA Health Care System, Durham, NC USA; 8 Department of Psychology, Georgetown University, Washington, DC USA; 9 Center for the Study of Aging and Human Development, Duke University Medical Center, Durham, NC USA; 10 Dunedin Multidisciplinary Health and Development Research Unit, Department of Psychology, University of Otago, Dunedin, New Zealand

**Keywords:** Genetics, Molecular biology

## Abstract

Epigenetic responses to cannabis use could link cannabis use to health problems. We examined the DNA-methylation profiles of long-term cannabis users in midlife, re-evaluating a set of 246 cannabis-associated methylation markers that were previously identified in other studies. Data were from the Dunedin Study, a five-decade longitudinal study of a birth cohort (analytic *n* = 787). Peripheral whole blood was drawn when the cohort was age 45, and DNA methylation was assayed using the EPIC 850 K BeadChip. Analyses compared long-term cannabis users with non-users and, for a benchmark comparison, long-term tobacco users. Results showed that long-term cannabis use was associated with sixteen of the previously published 246 cannabis-related methylation markers. Methylation markers that were associated with long-term cannabis use were also associated with long-term tobacco use. However, after adjusting for long-term tobacco use and other covariates, long-term cannabis use was robustly associated with hypomethylation of nine markers: cg05575921, cg21566642, cg03636183, cg21161138, cg01940273, cg17739917, cg05086879, cg02978227, cg23079012. Cannabis-related hypomethylation was associated with higher gene expression in the Dunedin Cohort, suggesting meaningful biological associations. A comparison of long-term cannabis users with cannabis quitters revealed that quitters showed less extreme DNA hypomethylation. Long-term cannabis use could affect the epigenome similarly to tobacco use, possibly at least partly though smoke inhalation. Cannabis cessation, like tobacco cessation, may reverse altered DNA methylation.

## Introduction

Long-term cannabis use has been linked to a number of health problems, including respiratory problems, cardiovascular diseases, cognitive decline, and mental illness [1]. Although some associations are inconsistent, evidence suggests that exposure to cannabinoids such as delta-9-tetrahydrocannabinol (THC) may pose risks to health. For example, cannabis administration studies have shown that THC, the main psychoactive constituent of cannabis, has acute effects on the vasculature [2] and on mental health symptoms [3]. Moreover, it is well known that smoking has negative effects on various aspects of health, and most people who use cannabis smoke it [4, 5]. These findings support the biological plausibility of effects of long-term cannabis use on some aspects of health.

Epigenetic responses to substance use, which can influence gene expression without altering genetic sequence, are increasingly recognized as mechanisms linking substance use to adverse health outcomes [6]. DNA methylation is one epigenetic mechanism, and it involves the addition or removal of methyl groups to DNA, usually at cytosine-guanine dinucleotides (CpG sites) [7]. Of all the substances, tobacco smoking has most consistently been associated with differential DNA methylation, notably hypomethylation of a CpG site located in the aryl hydrocarbon receptor repressor (*AHRR*) gene -- cg05575921 [8]. Hypomethylation of this site has been found in some studies to predict the development of lung cancer [9]. Importantly, hypomethylation of cg05575921 is linked to tobacco smoking and not non-combustible tobacco use [8], suggesting that it is exposure to the harmful chemicals in smoke, as opposed to nicotine, that causes the hypomethylation. The inhaled combustion products for cannabis and tobacco are largely similar [10], raising the question: do people who smoke cannabis show similar patterns of differential methylation to those who smoke tobacco?

The DNA methylation profiles of tobacco users have been fairly well characterized, but few studies have reported on the DNA methylation profiles of cannabis users. This represents a significant knowledge gap, especially given cannabis legalization and increases in cannabis use [11–15]. We identified eight studies published by August 2024 that reported on cannabis-related DNA methylation for up to eight hundred and fifty thousand CpG sites (Table S1) [16–23]. The studies generally found that cannabis use was associated with differential methylation of a small number of CpG sites after adjusting for multiple testing. However, the studies disagreed on which sites and on whether tobacco smoking accounted for cannabis associations. Moreover, some studies relied on small, unrepresentative samples. Most studies used relatively crude measures of cannabis use. For example, two studies distinguished only between those who had ever used versus never used cannabis [17, 23]. No studies examined whether cannabis cessation could restore DNA methylation profiles to that of non-users.

We aimed to redress these limitations in a representative birth cohort followed prospectively to midlife. We capitalized on the study’s strong and repeated assessments of cannabis use over a span of thirty years to identify, at midlife, a group of long-term cannabis users. Long-term cannabis users are of special clinical interest because long-term cannabis use is associated with more health, cognitive, and social problems compared with occasional, recreational use [1, 24–27]. Moreover, midlife is of interest because it is the developmental period when physical health decline becomes apparent [28] and when chronic diseases tend to onset [29].

This study addressed five questions. First, do previously reported cannabis-methylation associations replicate in a group of middle-age long-term cannabis users from an independent cohort? We elected a replication approach, as opposed to conducting an exploratory epigenome wide association study (EWAS), because EWAS requires very large samples to detect what are likely small-effect associations across hundreds of thousands of sites. We selected for analysis a set of 246 previously published cannabis-associated CpG sites (Table S2).

Second, how do long-term cannabis users compare with lifelong non-users and long-term tobacco users? We compared long-term cannabis users not only with non-users but also with long-term tobacco users, because many people who use cannabis also smoke tobacco and because the methylation profiles of long-term tobacco users are well characterized. Therefore, tobacco findings provide important context for interpreting cannabis findings. Analyses took two complementary approaches: (1) group comparisons of long-term cannabis users with lifelong non-users and long-term tobacco users, and (2) tests of dose-response associations, using, as the exposures, continuously-distributed quantitative measures of the extent of persistence of regular cannabis use and persistence of tobacco dependence over a lifetime. We elected to use a group comparison approach because it is the approach used in case-control studies. We elected to test dose-response associations because these tests are statistically powerful, and dose-response associations would be expected if associations were causal. Consistent findings across the two approaches would bolster confidence in cannabis-methylation associations.

Third, is differential DNA methylation among long-term cannabis users robust to control for confounders? We selected a set of plausible confounders and included them as covariates in tests of dose-response associations: childhood socioeconomic deprivation; low childhood self-control; family history of substance dependence; and persistence of tobacco, alcohol, and illicit drug use.

**Fig. 1:**
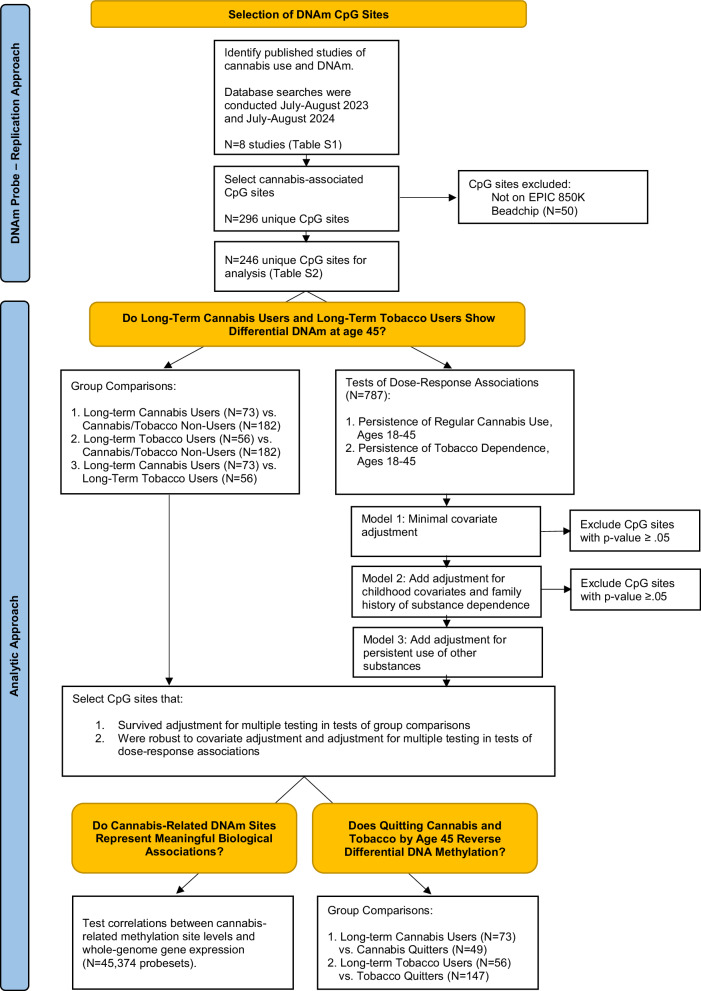
Flowchart documenting approach to CpG site selection and data analysis.

Fourth, do any of the cannabis-related methylation markers represent meaningful biological associations? Analyses examined correlations between cannabis-related methylation marker levels and whole-genome gene expression. Fifth, does cannabis cessation reverse differences in DNA methylation profiles? Analyses compared cannabis and tobacco quitters with non-users and with long-term cannabis users and long-term tobacco users. Our approach to addressing these questions is summarized in Fig. 1. Replicated findings of cannabis-related differential DNA methylation would suggest alterations in DNA methylation that could have important implications for later health.

## Methods

### Participants

Participants are members of the Dunedin Longitudinal Study, a representative birth cohort (*N* = 1,037; 91% of eligible births; 52% male) born April 1972-March 1973 in Dunedin, New Zealand (NZ), who were eligible based on residence in the province and who participated in the first assessment at age 3 years. As adults, the cohort’s distributions match the range in national surveys of same-age New Zealanders on educational attainment, body mass index, smoking, physical activity, and physician visits [30, 31]. Study participants are primarily of New Zealand European ethnicity; 8.6% reported Māori ethnicity at age 45.

Assessments were carried out at birth and ages 3, 5, 7, 9, 11, 13, 15, 18, 21, 26, 32, 38, and most recently (completed April 2019) 45 years. Of the original 1037 cohort participants, 997 were still alive at age 45; of these, 938 (94.1%) participated in the age-45 assessments [474 men (50.5%)]. Participants assessed at age 45 did not differ significantly from other living participants in terms of childhood SES, childhood IQ, or history of psychopathology (Fig. S1).

### Ethics approval and consent to participate

Participants gave written informed consent. Study protocols were approved by the NZ Health and Disability Ethics Committee (Protocol: 2017-1211). All methods were performed in accordance with the relevant guidelines and regulations.

### Measures

Measures are described here and more details are shown in Table S3.

#### Age-45 DNA methylation

At age 45, DNA extracted from peripheral whole blood was assayed using the EPIC 850 K BeadChip. Analyses are restricted to cohort members who did not self-identify as Māori at the time of the first blood draw and who consented to phlebotomy and whose DNA methylation passed quality control (*n* = 818).

As previously described [32], whole blood was collected in 10 mL K_2_EDTA tubes from 90% (*N* = 824) of participants at age 45. DNA was extracted from the buffy coat using standard procedures [33, 34]. ~500 ng of DNA from each sample was treated with sodium bisulfite using the EZ-96 DNA Methylation kit (Zymo Research, CA, USA). DNA methylation was quantified using the Illumina Infinium HumanMethylationEPIC BeadChip (“Illumina EPIC BeadChip”) run on an Illumina iScan System (Illumina, CA, USA) at the Molecular Genomics Core at the Duke Molecular Physiology Institute. All sample processing and array profiling was performed as a single experiment (i.e. at the same time).

Data were processed using ‘*wateRmelon’* (v1.26.0; [35]) and were normalized using the ‘*methylumi’* (v2.30.0; [36]) Bioconductor package from the R statistical programming environment and subjected to quality control (QC) analyses. Samples were removed if the average detection *p*-value was >=0.05. To confirm genetic identity of the DNA samples, we assessed genotype concordance between SNP probes on the EPIC array and data generated using Illumina OmniExpress12v1.1 genotyping BeadChips. Principal components analysis was performed on the full, normalized dataset and the first two components plotted [37]. Samples formed two major clusters separating on the 1st component, which corresponded to recorded sex. This was used to confirm sex assignment. Samples from 818 age-45 participants passed our QC pipeline.

To permit control for technical variation, we used residualized DNA methylation values for the principal components (PCs) using DNA methylation beta values for the 186 QC probes on the EPIC BeadChip. A total of 32 PCs were retained explaining 90% of the variation in QC probes. To control for cell type composition, we also included white cell-type counts measured using flow cytometry (Sysmex Corporation, Japan) in whole blood samples taken concurrently with the DNA sample.

#### Substance use

At ages 18, 21, 26, 32, 38, and 45, participants were interviewed about their substance use using the Diagnostic Interview Schedule [38, 39], and past-year substance-use dependencies were assessed following Diagnostic and Statistical Manual of Mental Disorders criteria. This information was used to define exposures for group comparisons and for tests of dose-response associations.

##### Groups for comparison

Groups comprised cohort members who met criteria at age 45 for (i) long-term cannabis use, (ii) long-term tobacco use, (ii) lifelong non-use of cannabis and tobacco, (iv) former cannabis use, (v) former tobacco use (Fig. S2). Subsets of cohort members who met criteria for these pre-registered groups were selected as described below and as previously published [24, 40, 41] to mimic groups from case-control studies. If a cohort member did not meet criteria for a group, they were not included in group comparisons (*n* = 332), but they were included in tests of dose-response associations. Table S4 shows background characteristics and substance use for the cohort and for each group described below.

*Long-term cannabis users* (*n* = 74; 68% male) used cannabis weekly or more frequently in the past year at age 45, or were dependent on cannabis at age 45, and also used weekly or more frequently at one or more previous assessment waves. Of these, 28% used cannabis before age 18; 74% met criteria for cannabis dependence at one or more waves; and 89% used regularly (4+ days per week) at one or more waves. Age-45 cannabis consumption was a median of 300 days in the past year, with 62% using 4+ days per week. Most long-term cannabis users had a history of tobacco dependence (84%) and smoked tobacco daily at age 45 (66%).

*Long-term tobacco users* (*n* = 57; 40% male) smoked tobacco daily at age 45 and also smoked daily at one or more previous waves; were free from cannabis at age 45; and had no history of weekly cannabis use or dependence.

*Lifelong cannabis/tobacco non-users* (*n* = 189; 40% male) never used cannabis, never used tobacco daily, and never had a diagnosis of any substance-use disorder.

*Cannabis quitters* (*n* = 50; 60% male) did not use cannabis at age 45 but previously either were diagnosed with cannabis dependence or used cannabis regularly (4+ days per week). Most had a history of tobacco dependence (60%) and a quarter smoked tobacco daily at age 45.

*Tobacco quitters* (*n* = 148; 45% male) did not use tobacco at age 45 but were previously diagnosed with tobacco dependence. Nearly a third of tobacco quitters had a history of cannabis dependence (29%), but few of them used cannabis regularly at age 45 (5%).

##### Quantitative exposures for tests of dose-response associations

We report on two previously published quantitative exposures [24, 40]: persistence of regular cannabis use and persistence of tobacco dependence.

*Persistence of regular cannabis use* (i.e., 4+ days per week) comprised participants who (i) never used cannabis (*n* = 244), (ii) used but never regularly (*n* = 450), (iii) used regularly at one study wave from age 18–45 years (*n* = 44), (iv) two waves (*n* = 26), (v) three waves (*n* = 26), and (vi) 4+ waves (*n* = 27).

*Persistence of tobacco dependence* comprised participants who (i) never smoked tobacco (*n* = 411), (ii) smoked tobacco daily at one or more assessment waves but were never diagnosed with tobacco dependence (*n* = 114), (iii) were diagnosed at one wave (*n* = 92), (iv) two waves (*n* = 81), (v) three waves (*n* = 52), and (vi) four or more waves (*n* = 67).

### Covariates

The following covariates were included in all statistical tests: sex, methylation array control probe principal components indexing technical variation, and white blood cell counts. White blood cell counts (neutrophils, lymphocytes, monocytes, eosinophils, and basophils) were included because there can be differences in patterns of methylation between different cell types, and people differ in the distribution of these cells in blood. The following additional covariates were included in tests of dose-response associations due to their association with cannabis use and/or DNA methylation: childhood socioeconomic status [42], low childhood self-control [43], family history of substance dependence [44], and persistent use of other substances (tobacco, alcohol, other illicit drugs) [45, 46].

### Statistical analyses

DNA methylation data were not normally distributed and showed evidence of outliers, influential observations, and, for some methylation markers, non-normally distributed residuals. Robust regression is appropriate when data contain outliers and influential observations. Further, research has shown that non-normally distributed residuals do not bias epigenome-wide association findings in terms of false negatives or false positives [47].

We used robust regression to compare groups on age-45 CpG sites, with groups represented by dummy codes. We used robust regression to test dose-response associations between quantitative exposures (persistence of regular cannabis use, persistence of tobacco dependence) and age-45 CpG sites. In tests of dose-response associations, covariates were added sequentially: sex, methylation array control probe principal components, and white blood cell counts (Model 1); aforementioned covariates plus childhood SES, low childhood self-control, and family history of substance dependence (Model 2); and aforementioned covariates plus persistent use of other substances (Model 3).

Prior to analyses, methylation probes were standardized on the full age-45 cohort (*N* = 818, M = 0.00, SD = 1.00). Standardized mean differences between groups can be interpreted as effect sizes, with 0.2, 0.5, 0.8 reflecting small, medium, and large effects, respectively [48]. Results present nominal *p*-values and indicate which CpG sites survived Bonferroni correction. All statistical tests are two-sided.

## Results

### A comparison of long-term cannabis users, long-term tobacco users, and non-users

Groups were compared on the 246 CpG sites shown in previous studies to be associated with cannabis use.

**Fig. 2:**
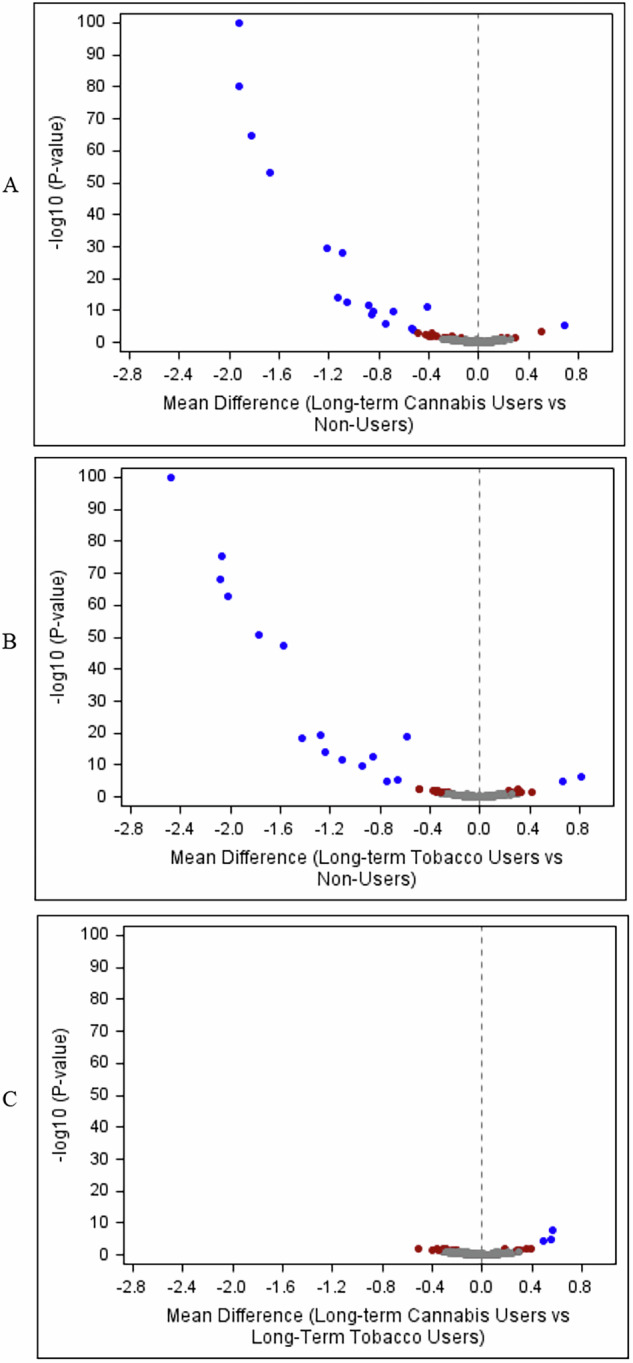
A comparison of long-term cannabis users, long-term tobacco users, and lifelong cannabis/tobacco non-users on age-45 DNA methylation. The x axis is the mean difference between long-term cannabis users and cannabis/tobacco non-users (Panel **A**), long-term tobacco users and cannabis/tobacco non-users (Panel **B**), and long-term cannabis users and long-term tobacco users (Panel **C**) on the 246 CpG sites at age 45. Mean differences were estimated using robust regression and were adjusted for sex, control probe principal components indexing technical variation, and white blood cell counts. Negative mean differences indicate hypomethylation relative to the comparison group, and positive mean differences indicate hypermethylation. The y-axis is the negative log base 10 p-value, and these were truncated at a value of 100. The dashed vertical line is the reference line that represents no difference in means between groups. Panel A=Long-term Cannabis Users (*N* = 73) vs. Cannabis/Tobacco Non-users (*N* = 182). Panel B=Long-term Tobacco Users (*N* = 56) vs. Cannabis/Tobacco Non-users (*N* = 182). Panel C=Long-term Cannabis Users (*N* = 73) vs. Long-term Tobacco Users (*N* = 56). The colors indicate statistical significance: Blue= *p* < 0.00020 (significant at Bonferroni-adjusted threshold for 246 tests). Red= *p* < 0.05. Gray= *p* ≥ 0.05.

Long-term cannabis users showed both hypomethylation and hypermethylation at age 45 relative to lifelong cannabis/tobacco non-users, but mostly hypomethylation (Fig. 2A). Of 246 CpG sites, 35 differed between long-term cannabis users and non-users at α = 0.05, and 17 at α adjusted for 246 tests (Table 1).

**Table 1:** Comparison of long-term cannabis users, long-term tobacco users, and cannabis/tobacco non-users on age-45 DNA methylation.

		Group Means (SD)						Long-Term Cannabis Users vs. Cannabis/Tobacco Non-Users				Long-Term Tobacco Users vs. Cannabis/Tobacco Non-Users				Long-Term Cannabis Users vs. Long-Term Tobacco Users			
		Lifelong Cannabis/Tobacco Non-Users (*N* = 182)		Long-Term Cannabis Users (*N* = 73)		Long-Term Tobacco Users (*N* = 56)		Adjusted Mean Difference	95% CI		*p*	Adjusted Mean Difference	95% CI		*p*	Adjusted Mean Difference	95% CI		*p*
	Age-45 CpG	M	SD	M	SD	M	SD												
1	cg05575921	0.57	0.17	−1.30	1.19	−1.64	1.01	−1.92	−2.08	−1.76	<0.0001^±^	−2.48	−2.65	−2.30	<0.0001^±^	0.56	0.37	0.76	<0.0001^±^
2	cg21566642	0.60	0.51	−1.12	1.03	−1.36	0.81	−1.92	−2.12	−1.72	<0.0001^±^	−2.06	−2.28	−1.84	<0.0001^±^	—	—	—	—
3	cg01940273	0.54	0.56	−1.11	0.99	−1.38	0.90	−1.82	−2.03	−1.61	<0.0001^±^	−2.07	−2.31	−1.84	<0.0001^±^	—	—	—	—
4	cg17739917	0.60	0.63	−1.13	0.96	−1.16	1.04	−1.67	−1.88	−1.46	<0.0001^±^	−2.02	−2.25	−1.78	<0.0001^±^	0.35	0.09	0.61	0.0084
5	cg21161138	0.46	0.46	−1.02	1.23	−1.38	1.02	−1.21	−1.42	−1.01	<0.0001^±^	−1.77	−2.00	−1.54	<0.0001^±^	0.56	0.30	0.82	<0.0001^±^
6	cg03636183	0.42	0.38	−1.17	1.39	−1.35	1.20	−1.09	−1.28	−0.89	<0.0001^±^	−1.58	−1.79	−1.37	<0.0001^±^	0.49	0.26	0.73	<0.0001^±^
7	cg25189904	0.37	0.79	−0.79	1.10	−0.95	1.01	−1.13	−1.41	−0.84	<0.0001^±^	−1.42	−1.74	−1.11	<0.0001^±^	—	—	—	—
8	cg18110140	0.38	0.78	−0.79	1.16	−0.89	1.02	−1.05	−1.33	−0.77	<0.0001^±^	−1.24	−1.56	−0.93	<0.0001^±^	—	—	—	—
9	cg05086879	0.34	0.60	−1.00	1.41	−1.14	1.15	−0.88	−1.12	−0.63	<0.0001^±^	−1.28	−1.55	−1.00	<0.0001^±^	0.40	0.10	0.70	0.0101
10	cg23079012	0.28	0.31	−0.77	1.77	−0.86	2.19	−0.41	−0.52	−0.29	<0.0001^±^	−0.59	−0.71	−0.46	<0.0001^±^	0.18	0.04	0.32	0.0125
11	cg02978227	0.27	0.58	−0.62	0.91	−0.67	0.79	−0.68	−0.89	−0.47	<0.0001^±^	−0.86	−1.09	−0.63	<0.0001^±^	—	—	—	—
12	cg18387338	0.19	0.84	−0.57	1.15	−0.78	0.93	−0.84	−1.10	−0.59	<0.0001^±^	−0.94	−1.23	−0.65	<0.0001^±^	—	—	—	—
13	cg09935388	0.27	0.74	−0.84	1.34	−0.96	1.31	−0.86	−1.14	−0.58	<0.0001^±^	−1.10	−1.41	−0.80	<0.0001^±^	—	—	—	—
14	cg23916896	0.26	1.01	−0.42	1.03	−0.58	0.73	−0.74	−1.04	−0.44	<0.0001^±^	−0.75	−1.08	−0.41	<0.0001^±^	—	—	—	—
15	cg05009104	−0.22	0.92	0.47	0.85	0.63	0.91	0.69	0.40	0.98	<0.0001^±^	0.82	0.50	1.14	<0.0001^±^	—	—	—	—
16	cg26286198	0.17	0.88	−0.37	0.91	−0.38	1.12	−0.54	−0.80	−0.28	0.0001^±^	−0.36	−0.65	−0.08	0.0136	—	—	—	—
17	cg15088912	0.12	0.93	−0.36	1.16	−0.68	1.14	−0.52	−0.77	−0.26	0.0001^±^	−0.65	−0.94	−0.37	0.0000^±^	—	—	—	—
18	cg19089201	−0.20	1.02	0.37	0.78	0.63	0.83	0.51	0.23	0.78	0.0003	0.67	0.37	0.97	0.0000^±^	—	—	—	—
19	cg17350345	−0.04	1.06	−0.26	0.98	−0.13	1.00	−0.38	−0.60	−0.15	0.0010	−0.33	−0.58	−0.08	0.0094	—	—	—	—
20	cg26764244	0.14	0.96	−0.34	1.01	−0.39	0.94	−0.48	−0.78	−0.19	0.0013	−0.48	−0.81	−0.16	0.0037	—	—	—	—
21	cg22124000	0.08	0.91	−0.18	1.08	−0.52	1.20	−0.43	−0.72	−0.14	0.0035	−0.49	−0.81	−0.17	0.0026	—	—	—	—
22	cg14219071	0.06	1.07	−0.22	0.93	−0.12	0.86	−0.40	−0.69	−0.11	0.0062	—	—	—	—	—	—	—	—
23	cg12510044	0.10	1.00	−0.26	0.94	−0.35	1.05	−0.37	−0.64	−0.09	0.0084	−0.35	−0.65	−0.04	0.0255	—	—	—	—
24	cg21852554	−0.13	1.07	−0.05	1.02	0.19	0.83	−0.21	−0.36	−0.05	0.0086	—	—	—	—	−0.21	−0.40	−0.02	0.0284
25	cg16276224	0.07	0.91	−0.28	1.02	−0.09	1.05	−0.39	−0.68	−0.09	0.0108	—	—	—	—	—	—	—	—
26	cg21491555	0.11	0.91	−0.17	0.95	−0.12	1.29	−0.34	−0.61	−0.07	0.0143	—	—	—	—	—	—	—	—
27	cg15802887	−0.18	1.05	−0.02	1.01	0.10	0.88	−0.14	−0.26	−0.02	0.0192	—	—	—	—	—	—	—	—
28	cg13966609	−0.04	0.97	0.14	0.86	0.18	0.77	0.18	0.02	0.34	0.0254	—	—	—	—	—	—	—	—
29	cg18880190	−0.02	0.91	−0.29	1.05	−0.28	0.93	−0.28	−0.52	−0.03	0.0288	−0.37	−0.64	−0.10	0.0083	—	—	—	—
30	cg22572071	0.08	0.95	−0.29	1.16	−0.35	1.04	−0.27	−0.51	−0.03	0.0294	—	—	—	—	—	—	—	—
31	cg04864586	0.06	0.89	−0.03	0.82	0.20	0.73	−0.20	−0.38	−0.02	0.0314	—	—	—	—	−0.31	−0.54	−0.09	0.0060
32	cg05922265	−0.05	0.99	0.25	0.73	0.02	0.75	0.24	0.02	0.45	0.0337	—	—	—	—	—	—	—	—
33	cg03093806	0.12	1.03	−0.19	0.95	−0.27	0.82	−0.27	−0.53	−0.02	0.0357	—	—	—	—	—	—	—	—
34	cg27055782	0.07	0.87	−0.24	0.80	−0.22	1.89	−0.27	−0.52	−0.02	0.0376	—	—	—	—	—	—	—	—
35	cg08153621	−0.07	1.03	0.21	0.86	0.24	1.06	0.29	0.02	0.56	0.0377	—	—	—	—	—	—	—	—
36	cg00813162	−0.16	0.95	0.08	0.98	0.42	0.96	—	—	—	—	0.30	0.10	0.51	0.0039	—	—	—	—
37	cg27209861	−0.24	1.07	0.11	0.91	0.22	0.90	—	—	—	—	0.24	0.05	0.42	0.0132	—	—	—	—
38	cg11175241	−0.13	1.05	−0.05	1.14	0.30	0.92	—	—	—	—	0.25	0.04	0.45	0.0194	−0.29	−0.52	−0.06	0.0140
39	cg08688629	0.05	1.07	−0.17	0.87	0.37	1.03	—	—	—	—	0.41	0.07	0.76	0.0197	−0.51	−0.90	−0.12	0.0100
40	cg17464820	−0.03	0.99	−0.04	1.03	−0.03	0.98	—	—	—	—	−0.29	−0.54	−0.04	0.0244	—	—	—	—
41	cg15204119	0.09	0.92	−0.04	0.94	−0.30	0.87	—	—	—	—	−0.28	−0.53	−0.03	0.0267	—	—	—	—
42	cg08839808	−0.19	1.05	0.05	0.94	0.36	0.69	—	—	—	—	0.26	0.03	0.48	0.0274	—	—	—	—
43	cg03802952	0.12	0.98	−0.21	0.97	−0.38	1.00	—	—	—	—	−0.31	−0.59	−0.03	0.0281	—	—	—	—
44	cg02235741	0.07	1.03	0.02	0.86	−0.16	1.03	—	—	—	—	−0.32	−0.60	−0.03	0.0301	—	—	—	—
45	cg10328583	−0.11	0.97	0.14	1.00	0.28	0.98	—	—	—	—	0.30	0.03	0.57	0.0321	—	—	—	—
46	cg17250160	−0.21	0.94	0.12	1.05	0.33	1.11	—	—	—	—	0.33	0.02	0.64	0.0362	—	—	—	—
47	cg12028375	0.04	0.75	−0.07	0.74	−0.14	0.80	—	—	—	—	−0.24	−0.48	−0.01	0.0417	—	—	—	—
48	cg10620881	−0.09	1.12	0.22	0.91	0.25	0.68	—	—	—	—	0.22	0.00	0.44	0.0498	—	—	—	—
49	cg09501516	−0.03	0.84	−0.14	0.72	0.09	1.00	—	—	—	—	—	—	—	—	−0.37	−0.63	−0.10	0.0076
50	cg21960184	0.00	0.97	−0.08	1.08	0.10	0.93	—	—	—	—	—	—	—	—	−0.40	−0.76	−0.04	0.0316
51	cg09825346	−0.04	0.92	0.22	0.85	−0.11	0.71	—	—	—	—	—	—	—	—	0.28	0.02	0.54	0.0322
52	cg25069772	0.03	0.63	0.04	0.64	0.15	0.73	—	—	—	—	—	—	—	—	−0.20	−0.40	−0.01	0.0430
53	cg21998512	0.03	0.98	0.08	0.99	−0.11	0.96	—	—	—	—	—	—	—	—	0.31	0.01	0.61	0.0452
54	cg00250546	0.02	1.00	0.00	0.99	0.03	1.36	—	—	—	—	—	—	—	—	−0.24	−0.48	0.00	0.0473
55	cg20958467	−0.08	1.00	−0.03	1.01	0.16	0.96	—	—	—	—	—	—	—	—	−0.35	−0.69	0.00	0.0479

Results are shown for the CpG sites that were statistically significant at α = 0.05 (long-term cannabis users vs. non-users [*n* = 35 CpG sites]; long-term tobacco users vs. non-users [*n* = 36 CpG sites]; and long-term cannabis users vs. long-term tobacco users [*n* = 17 CpG sites]). Means are crude means, unadjusted for covariates, and standardized (M = 0.00, SD = 1.00) on the representative cohort. Adjusted mean differences are mean differences from robust regression, adjusted for sex, methylation-array control probe principal components indexing technical variation, and white blood cell counts. CpG sites are ordered by the *p*-value for the comparison of long-term cannabis users with non-users, and then by the *p*-value for the comparison of long-term tobacco users with non-users, and finally by the *p*-value for the comparison of long-term cannabis users with long-term tobacco users. Dashes=not statistically significant at α = 0.05. Group Ns are slightly reduced due to missing covariate data for 9 cohort members.

^±^statistically significant at α adjusted for 246 tests.

Long-term tobacco users showed both hypomethylation and hypermethylation at age 45 compared with lifelong cannabis/tobacco non-users, but mostly hypomethylation (Fig. 2B). Of 246 CpG sites, 36 differed between long-term tobacco users and non-users at α = 0.05, and 17 at α adjusted for 246 tests (Table 1).

Long-term cannabis users and long-term tobacco users generally did not differ in terms of age-45 DNA methylation (Fig. 2C). Of 246 CpG sites, 17 differed between long-term cannabis users and long-term tobacco users at α = 0.05, and 3 at α adjusted for 246 tests (Table 1).

### Tests of dose-response associations for persistence of regular cannabis use and persistence of tobacco dependence

Greater persistence of regular cannabis use from age 18–45 was associated with differential DNA methylation of 52 of 246 CpG sites at α = 0.05, and 17 at α adjusted for 246 tests (Table 2, Model 1). Associations were largely unchanged after adjusting for childhood SES, low childhood self-control, and family history of substance dependence, with 45 of the 52 significant CpG sites from Model 1 remaining differentially methylated at α = 0.05, and 15 at α adjusted for 52 tests (Table 2, Model 2). A number of associations decreased substantially after additionally adjusting for persistence of tobacco, alcohol, and illicit drug use. Nonetheless, 19 of the 45 significant CpG sites from Model 2 remained differentially methylated at α = 0.05 after adjusting for all covariates, and 9 of these were significant at α adjusted for 45 tests (Table 2, Model 3).

**Table 2:** Dose-response associations between persistence of regular cannabis use from age 18–45 and age-45 DNA methylation.

		Model 1: Adjusted for Sex, PCs, and White Blood Cell Counts (*N* = 787)				Model 2: + Adjustment for Childhood SES, Childhood Low Self-Control, and Family History of Substance Dependence (*N* = 787)				Model 3: + Adjustment for Persistence of Tobacco Dependence, Persistence of Alcohol Dependence, and Persistent Illicit Drug Dependence (*N* = 787)			
	Age-45 CpG	Est	95% CI		*p*	Est	95% CI		*p*	Est	95% CI		*p*
1	cg05575921	−0.30	−0.33	−0.27	<0.0001^±^	−0.30	−0.33	−0.28	<0.0001^±^	−0.15	−0.18	−0.12	<0.0001^±^
2	cg21566642	−0.40	−0.45	−0.34	<0.0001^±^	−0.36	−0.42	−0.31	<0.0001^±^	−0.15	−0.20	−0.09	<0.0001^±^
3	cg03636183	−0.27	−0.30	−0.23	<0.0001^±^	−0.25	−0.29	−0.21	<0.0001^±^	−0.14	−0.18	−0.10	<0.0001^±^
4	cg21161138	−0.27	−0.31	−0.23	<0.0001^±^	−0.26	−0.31	−0.22	<0.0001^±^	−0.14	−0.19	−0.09	<0.0001^±^
5	cg01940273	−0.35	−0.41	−0.30	<0.0001^±^	−0.32	−0.38	−0.27	<0.0001^±^	−0.14	−0.20	−0.08	<0.0001^±^
6	cg17739917	−0.33	−0.38	−0.28	<0.0001^±^	−0.31	−0.36	−0.26	<0.0001^±^	−0.16	−0.22	−0.11	<0.0001^±^
7	cg05086879	−0.24	−0.29	−0.20	<0.0001^±^	−0.23	−0.27	−0.18	<0.0001^±^	−0.15	−0.21	−0.10	<0.0001^±^
8	cg09935388	−0.19	−0.23	−0.15	<0.0001^±^	−0.15	−0.19	−0.11	<0.0001^±^	−0.07	−0.11	−0.02	0.0027
9	cg02978227	−0.18	−0.22	−0.13	<0.0001^±^	−0.16	−0.20	−0.11	<0.0001^±^	−0.09	−0.14	−0.04	0.0003^±^
10	cg23079012	−0.07	−0.09	−0.05	<0.0001^±^	−0.06	−0.08	−0.05	<0.0001^±^	−0.05	−0.07	−0.02	<0.0001^±^
11	cg25189904	−0.22	−0.29	−0.16	<0.0001^±^	−0.19	−0.26	−0.13	<0.0001^±^	−0.07	−0.14	0.00	0.0586
12	cg18110140	−0.20	−0.26	−0.14	<0.0001^±^	−0.17	−0.24	−0.11	<0.0001^±^	−0.05	−0.11	0.02	0.1449
13	cg23916896	−0.18	−0.24	−0.11	<0.0001^±^	−0.15	−0.22	−0.08	<0.0001^±^	−0.06	−0.14	0.01	0.1038
14	cg15088912	−0.14	−0.20	−0.09	<0.0001^±^	−0.13	−0.18	−0.07	<0.0001^±^	−0.06	−0.12	0.01	0.0922
15	cg05009104	0.14	0.07	0.21	<0.0001^±^	0.11	0.04	0.19	0.0021	0.02	−0.06	0.10	0.6140
16	cg05208619	−0.12	−0.17	−0.06	0.0002^±^	−0.11	−0.17	−0.04	0.0008^±^	−0.10	−0.17	−0.03	0.0065
17	cg18387338	−0.11	−0.17	−0.05	0.0002^±^	−0.10	−0.16	−0.04	0.0011	−0.02	−0.08	0.05	0.6267
18	cg19089201	0.12	0.06	0.19	0.0003	0.10	0.03	0.16	0.0049	0.02	−0.06	0.09	0.6228
19	cg26286198	−0.10	−0.16	−0.05	0.0004	−0.10	−0.16	−0.04	0.0012	−0.09	−0.16	−0.02	0.0086
20	cg22124000	−0.11	−0.17	−0.05	0.0006	−0.10	−0.16	−0.03	0.0025	−0.05	−0.12	0.03	0.2082
21	cg05161803	−0.09	−0.14	−0.04	0.0009	−0.08	−0.13	−0.03	0.0038	−0.06	−0.12	0.00	0.0441
22	cg23268344	−0.10	−0.16	−0.04	0.0010	−0.10	−0.16	−0.04	0.0011	−0.10	−0.17	−0.03	0.0071
23	cg02616769	−0.10	−0.16	−0.04	0.0011	−0.09	−0.15	−0.03	0.0030	−0.09	−0.16	−0.02	0.0114
24	cg22572071	−0.08	−0.13	−0.03	0.0025	−0.06	−0.12	−0.01	0.0192	−0.03	−0.09	0.03	0.2907
25	cg13810485	−0.07	−0.12	−0.02	0.0042	−0.06	−0.11	−0.01	0.0237	−0.04	−0.10	0.01	0.1420
26	cg16276224	−0.08	−0.14	−0.02	0.0059	−0.08	−0.15	−0.02	0.0084	−0.05	−0.12	0.02	0.1644
27	cg00761236	−0.07	−0.11	−0.02	0.0066	−0.06	−0.11	−0.01	0.0214	−0.02	−0.08	0.03	0.4236
28	cg17218147	−0.07	−0.12	−0.02	0.0075	−0.06	−0.11	−0.01	0.0175	−0.06	−0.12	0.00	0.0591
29	cg08153621	0.08	0.02	0.14	0.0091	0.06	0.00	0.13	0.0420	0.03	−0.04	0.10	0.4224
30	cg10620881	0.06	0.01	0.10	0.0104	0.05	0.01	0.10	0.0185	0.05	0.00	0.10	0.0513
31	cg14219071	−0.08	−0.14	−0.02	0.0107	−0.07	−0.13	−0.01	0.0234	−0.03	−0.10	0.04	0.3522
32	cg23932689	−0.07	−0.12	−0.01	0.0113	−0.06	−0.11	−0.01	0.0238	−0.06	−0.12	0.01	0.0726
33	cg17350345	−0.06	−0.11	−0.01	0.0114	−0.06	−0.11	−0.01	0.0234	−0.02	−0.08	0.03	0.4047
34	cg01212491	−0.07	−0.12	−0.02	0.0116	−0.06	−0.11	0.00	0.0417	−0.03	−0.09	0.03	0.3641
35	cg17250160	0.08	0.02	0.14	0.0118	0.06	0.00	0.13	0.0400	0.06	−0.01	0.13	0.1050
36	cg26764244	−0.08	−0.14	−0.02	0.0149	−0.06	−0.12	0.01	0.0837	—	—	—	—
37	cg14374923	−0.07	−0.13	−0.01	0.0151	−0.06	−0.13	0.00	0.0390	−0.10	−0.17	−0.02	0.0083
38	cg06815210	−0.07	−0.13	−0.01	0.0168	−0.07	−0.13	−0.01	0.0297	−0.08	−0.15	−0.01	0.0185
39	cg03802952	−0.06	−0.11	−0.01	0.0169	−0.05	−0.10	0.00	0.0685	—	—	—	—
40	cg23314514	−0.07	−0.13	−0.01	0.0189	−0.08	−0.14	−0.02	0.0111	−0.06	−0.13	0.01	0.0788
41	cg03905975	−0.07	−0.13	−0.01	0.0206	−0.07	−0.13	0.00	0.0349	−0.07	−0.14	0.00	0.0563
42	cg13179084	0.03	0.00	0.06	0.0221	0.03	0.01	0.06	0.0189	0.03	0.00	0.07	0.0512
43	cg12510044	−0.06	−0.12	−0.01	0.0222	−0.06	−0.12	0.00	0.0432	−0.02	−0.08	0.05	0.5910
44	cg01039752	−0.07	−0.13	−0.01	0.0222	−0.08	−0.14	−0.02	0.0117	−0.07	−0.15	0.00	0.0454
45	cg01668099	−0.06	−0.11	−0.01	0.0225	−0.05	−0.10	0.00	0.0713	−	−	−	−
46	cg24976193	−0.06	−0.11	−0.01	0.0242	−0.05	−0.10	0.00	0.0680	—	—	—	—
47	cg07011775	−0.06	−0.12	−0.01	0.0250	−0.06	−0.12	0.00	0.0488	−0.07	−0.14	0.00	0.0436
48	cg03093806	−0.07	−0.12	−0.01	0.0254	−0.06	−0.12	0.00	0.0622	—	—	—	—
49	cg00785657	0.08	0.01	0.15	0.0271	0.06	−0.01	0.13	0.0845	—	—	—	—
50	cg09706133	−0.06	−0.11	−0.01	0.0314	−0.04	−0.10	0.01	0.1032	—	—	—	—
51	cg19856593	−0.07	−0.13	−0.01	0.0339	−0.08	−0.14	−0.01	0.0219	−0.06	−0.14	0.02	0.1196
52	cg04864586	−0.04	−0.09	0.00	0.0352	−0.05	−0.09	0.00	0.0341	−0.03	−0.08	0.02	0.1961

Results are shown for CpG sites that were statistically significant at α = 0.05 in Model 1, the minimally-adjusted model (*n* = 52 CpG sites). All associations are adjusted for sex, methylation-array control probe principal components indexing technical variation, and white blood cell counts. CpG sites are ordered by the *p*-value in Model 1. Est=standardized beta coefficient from robust regression. Dashes=not tested.

*PCs* principal components indexing technical variation, *SES* socioeconomic status.

^±^Statistically significant after adjustment for multiple testing. Analytic N is slightly reduced due to missing covariate data.

Greater persistence of tobacco dependence was associated with differential methylation of 57 of the 246 CpG sites at α = 0.05, and 22 at α adjusted for 246 tests (Table 3, Model 1). Associations were largely unchanged after adjusting for childhood SES, low childhood self-control, and family history of substance dependence, with 47 of the 57 significant CpG sites from Model 1 remaining differentially methylated at α = 0.05, and 22 at α adjusted for 57 tests (Table 3, Model 2). Associations were only slightly attenuated after adjusting for persistence of cannabis, alcohol, and illicit drug use, with 37 of the 47 significant CpG sites from Model 2 remaining differentially methylated at α = 0.05 after adjustment for all covariates, and 20 of these were significant at α adjusted for 47 tests (Table 3, Model 3).

**Table 3:** Dose-response associations between persistence of tobacco dependence from age 18–45 and age-45 DNA methylation.

		Model 1: Adjusted for Sex, PCs, and White Blood Cell Counts (*N* = 787)				Model 2: + Adjustment for Childhood SES, Childhood Low Self-Control, Family History of Substance Dependence (*N* = 787)				Model 3: + Adjustment for Persistence of Regular Cannabis Use, Persistence of Alcohol Dependence, Persistent Illicit Drug Dependence (*N* = 787)			
	Age-45 CpG	Est	95% CI		*p*	Est	95% CI		*p*	Est	95% CI		*p*
1	cg05575921^a^	−0.26	−0.28	−0.24	<0.0001^±^	−0.26	−0.28	−0.24	<0.0001^±^	−0.22	−0.24	−0.20	<0.0001^±^
2	cg21566642^a^	−0.38	−0.41	−0.34	<0.0001^±^	−0.36	−0.40	−0.33	<0.0001^±^	−0.32	−0.36	−0.29	<0.0001^±^
3	cg01940273^a^	−0.35	−0.38	−0.32	<0.0001^±^	−0.34	−0.37	−0.30	<0.0001^±^	−0.30	−0.34	−0.27	<0.0001^±^
4	cg03636183^a^	−0.25	−0.28	−0.23	<0.0001^±^	−0.25	−0.27	−0.22	<0.0001^±^	−0.21	−0.24	−0.18	<0.0001^±^
5	cg17739917^a^	−0.30	−0.34	−0.27	<0.0001^±^	−0.30	−0.34	−0.27	<0.0001^±^	−0.26	−0.30	−0.23	<0.0001^±^
6	cg21161138^a^	−0.23	−0.26	−0.20	<0.0001^±^	−0.23	−0.25	−0.20	<0.0001^±^	−0.19	−0.22	−0.16	<0.0001^±^
7	cg18110140^a^	−0.26	−0.29	−0.22	<0.0001^±^	−0.25	−0.28	−0.21	<0.0001^±^	−0.24	−0.28	−0.20	<0.0001^±^
8	cg09935388^a^	−0.17	−0.20	−0.15	<0.0001^±^	−0.16	−0.18	−0.13	<0.0001^±^	−0.14	−0.16	−0.11	<0.0001^±^
9	cg25189904^a^	−0.26	−0.30	−0.21	<0.0001^±^	−0.25	−0.29	−0.21	<0.0001^±^	−0.23	−0.28	−0.18	<0.0001^±^
10	cg05086879^a^	−0.19	−0.22	−0.16	<0.0001^±^	−0.18	−0.21	−0.15	<0.0001^±^	−0.15	−0.18	−0.11	<0.0001^±^
11	cg02978227^a^	−0.16	−0.19	−0.13	<0.0001^±^	−0.15	−0.18	−0.12	<0.0001^±^	−0.13	−0.16	−0.10	<0.0001^±^
12	cg23079012^a^	−0.06	−0.07	−0.04	<0.0001^±^	−0.05	−0.06	−0.04	<0.0001^±^	−0.04	−0.06	−0.03	<0.0001^±^
13	cg05009104^a^	0.19	0.15	0.24	<0.0001^±^	0.18	0.13	0.23	<0.0001^±^	0.18	0.13	0.23	<0.0001^±^
14	cg23916896^a^	−0.18	−0.23	−0.14	<0.0001^±^	−0.17	−0.22	−0.13	<0.0001^±^	−0.16	−0.21	−0.11	<0.0001^±^
15	cg18387338^a^	−0.15	−0.19	−0.12	<0.0001^±^	−0.15	−0.19	−0.11	<0.0001^±^	−0.14	−0.18	−0.10	<0.0001^±^
16	cg15088912^a^	−0.14	−0.18	−0.11	<0.0001^±^	−0.14	−0.17	−0.10	<0.0001^±^	−0.12	−0.16	−0.08	<0.0001^±^
17	cg19089201^a^	0.15	0.11	0.19	<0.0001^±^	0.14	0.09	0.18	<0.0001^±^	0.14	0.09	0.19	<0.0001^±^
18	cg26764244	−0.13	−0.17	−0.09	<0.0001^±^	−0.12	−0.16	−0.07	<0.0001^±^	−0.12	−0.16	−0.07	<0.0001^±^
19	cg22124000	−0.12	−0.16	−0.08	<0.0001^±^	−0.11	−0.16	−0.07	<0.0001^±^	−0.10	−0.15	−0.05	<0.0001^±^
20	cg17350345	−0.06	−0.09	−0.03	<0.0001^±^	−0.06	−0.10	−0.03	0.0002^±^	−0.06	−0.09	−0.02	0.0019
21	cg18880190	−0.07	−0.11	−0.03	0.0001^±^	−0.07	−0.11	−0.03	0.0003^±^	−0.07	−0.11	−0.02	0.0023
22	cg22112841	0.07	0.03	0.11	0.0001^±^	0.07	0.04	0.11	0.0001^±^	0.09	0.05	0.13	<0.0001^±^
23	cg01101459	0.07	0.03	0.10	0.0005	0.05	0.01	0.09	0.0138	0.06	0.02	0.10	0.0073
24	cg17250160	0.07	0.03	0.11	0.0006	0.06	0.02	0.10	0.0043	0.06	0.02	0.11	0.0063
25	cg26870460	0.07	0.03	0.11	0.0007	0.07	0.03	0.11	0.0014	0.07	0.02	0.11	0.0034
26	cg27209861	0.04	0.02	0.06	0.0007	0.03	0.01	0.06	0.0058	0.04	0.01	0.06	0.0046
27	cg14219071	−0.07	−0.11	−0.03	0.0011	−0.06	−0.11	−0.02	0.0026	−0.05	−0.10	−0.01	0.0265
28	cg12510044	−0.06	−0.10	−0.02	0.0012	−0.06	−0.10	−0.02	0.0027	−0.04	−0.09	0.00	0.0411
29	cg22572071	−0.06	−0.09	−0.02	0.0013	−0.04	−0.08	−0.01	0.0183	−0.03	−0.07	0.01	0.2038
30	cg03093806	−0.06	−0.10	−0.02	0.0015	−0.06	−0.10	−0.02	0.0050	−0.05	−0.10	−0.01	0.0195
31	cg00813162	0.04	0.02	0.07	0.0016	0.04	0.01	0.07	0.0074	0.05	0.02	0.08	0.0018
32	cg01668099	−0.05	−0.09	−0.02	0.0019	−0.04	−0.08	−0.01	0.0137	−0.03	−0.07	0.01	0.0893
33	cg01212491	−0.05	−0.09	−0.02	0.0049	−0.04	−0.08	−0.01	0.0214	−0.03	−0.07	0.01	0.1263
34	cg13966609	0.03	0.01	0.06	0.0055	0.03	0.01	0.06	0.0114	0.03	0.01	0.06	0.0146
35	cg02337960	−0.05	−0.08	−0.01	0.0068	−0.06	−0.09	−0.02	0.0031	−0.05	−0.09	−0.01	0.0128
36	cg15204119	−0.05	−0.09	−0.01	0.0080	−0.05	−0.09	−0.01	0.0159	−0.05	−0.09	0.00	0.0404
37	cg03802952	−0.05	−0.08	−0.01	0.0085	−0.04	−0.07	0.00	0.0556	—	—	—	—
38	cg00761236	−0.04	−0.07	−0.01	0.0103	−0.03	−0.07	0.00	0.0420	−0.02	−0.06	0.02	0.3042
39	cg05161803	−0.04	−0.08	−0.01	0.0111	−0.03	−0.07	0.00	0.0576	—	—	—	—
40	cg16276224	−0.05	−0.09	−0.01	0.0130	−0.04	−0.09	0.00	0.0407	−0.03	−0.07	0.02	0.2444
41	cg10328583	0.05	0.01	0.08	0.0137	0.04	0.00	0.07	0.0702	—	—	—	—
42	cg08153621	0.05	0.01	0.09	0.0146	0.04	0.00	0.09	0.0444	0.03	−0.02	0.07	0.2761
43	cg19856593	−0.05	−0.10	−0.01	0.0148	−0.06	−0.10	−0.02	0.0084	−0.04	−0.09	0.01	0.1099
44	cg10620881	0.04	0.01	0.07	0.0190	0.03	0.00	0.06	0.0406	0.02	−0.01	0.06	0.1814
45	cg01806956	0.04	0.01	0.07	0.0223	0.04	0.00	0.08	0.0268	0.05	0.01	0.09	0.0131
46	cg12710152	−0.05	−0.09	−0.01	0.0240	−0.04	−0.09	0.00	0.0357	−0.05	−0.10	−0.01	0.0269
47	cg24007376	0.03	0.00	0.06	0.0241	0.03	0.01	0.06	0.0165	0.03	0.00	0.06	0.0401
48	cg25228746	0.04	0.00	0.08	0.0261	0.03	−0.01	0.07	0.1323	—	—	—	—
49	cg15425280	0.04	0.01	0.08	0.0268	0.04	0.00	0.08	0.0448	0.04	−0.01	0.08	0.1146
50	cg13810485	−0.04	−0.07	0.00	0.0268	−0.03	−0.06	0.01	0.1437	—	—	—	—
51	cg02705374	−0.04	−0.07	0.00	0.0302	−0.03	−0.07	0.00	0.0840	—	—	—	—
52	cg26286198	−0.04	−0.08	0.00	0.0321	−0.04	−0.08	0.00	0.0845	—	—	—	—
53	cg04904300	0.01	0.00	0.02	0.0330	0.01	0.00	0.02	0.0427	0.01	0.00	0.02	0.0220
54	cg11175241	0.03	0.00	0.06	0.0333	0.03	0.00	0.05	0.0623	—	—	—	—
55	cg04831510	−0.04	−0.08	0.00	0.0341	−0.04	−0.08	0.00	0.0608	—	—	—	—
56	cg19772897	−0.04	−0.07	0.00	0.0369	−0.03	−0.06	0.01	0.1104	—	—	—	—
57	cg09825346	0.03	0.00	0.05	0.0475	0.03	0.00	0.06	0.0488	0.03	0.00	0.06	0.0704

Results are shown for the CpG sites that were statistically significant at α = 0.05 in Model 1, the minimally-adjusted model (*n* = 57 CpG sites). All associations are adjusted for sex, methylation-array control probe principal components indexing technical variation, and white blood cell counts. CpG sites are ordered by the *p*-value in Model 1. Est=standardized beta coefficient from robust regression. Dashes=not tested.

*PCs* principal components indexing technical variation, *SES* socioeconomic status.

^±^Statistically significant after adjustment for multiple testing.

^a^CpG site was statistically significant after adjustment for multiple testing in analyses comparing long-term tobacco users with non-users (Table 1) and in Model 1 tests of dose-response associations (Table 3). Analytic N is slightly reduced due to missing covariate data.

### Summary of group comparisons and tests of dose-response associations

Table S5 summarizes the cannabis-related CpGs from tests of group comparisons and tests of dose-response associations. Of the 246 candidate CpG sites that we sought to replicate, 35 replicated in tests comparing long-term cannabis users with non-users and 52 replicated in tests of persistence of regular cannabis use, using comparable covariates across the two approaches (sex, control probe principal components indexing technical variation, white blood cell counts) and using α = 0.05. After adjusting for multiple testing, those numbers reduced to 17 (Table 1) and 17 (Table 2, Model 1) CpG sites, respectively, with 16 CpGs in common across the two approaches. When dose-response associations were additionally adjusted for substantive covariates, including childhood SES, low childhood self-control, family history of substance dependence, and persistence of tobacco, alcohol, and illicit drug use, nine CpGs remained associated with persistence of regular cannabis use at α adjusted for multiple testing (Table 2, Model 3: cg05575921, cg21566642, cg03636183, cg21161138, cg01940273, cg17739917, cg05086879, cg02978227, cg23079012). By comparison, there were 17 tobacco-related CpG sites that survived adjustment for multiple testing in group comparisons and were robust to covariate-adjustment and adjustment for multiple testing in tests of dose-response associations. The 9 cannabis-related CpG sites that replicated in tests of group comparisons and covariate-adjusted tests of dose-response associations were the focus of the next two analyses: (1) a test of associations between methylation sites and gene expression, and (2) a test of whether quitters showed less extreme hypomethylation than long-term cannabis users.

### Association between DNA methylation and gene expression levels

To test whether the nine cannabis-related methylation sites represent meaningful biological associations, we examined correlations between methylation site levels and whole-genome gene expression (*N* = 45,374 probesets). Methods are described in Supplemental Text. Gene expression was measured when study members were 38 years old. Therefore, these analyses used gene expression and DNA methylation data collected concurrently at age 38. Because DNA methylation data were assayed using different arrays at age 38 and age 45, three of the nine cannabis-related CpG sites at age 45 were not present in the age-38 data. Of the remaining six CpG sites (cg05575921, cg21566642, cg03636183, cg21161138, cg01940273, cg23079012), all were associated with long-term cannabis use at age 38, like at age 45 (Table S6), and all showed statistically significant negative associations with gene expression levels after adjustment for multiple testing (Fig. S3). Specifically, across the six cannabis-related CpG sites, hypomethylation was associated with higher expression of *AHRR, P2RY6, LRRN3, GPR15*, and *DSC2*. Two sites were at the *AHRR* locus (cg05575921 and cg21161138).

### A comparison of cannabis and tobacco quitters with non-users and long-term users

**Fig. 3:**
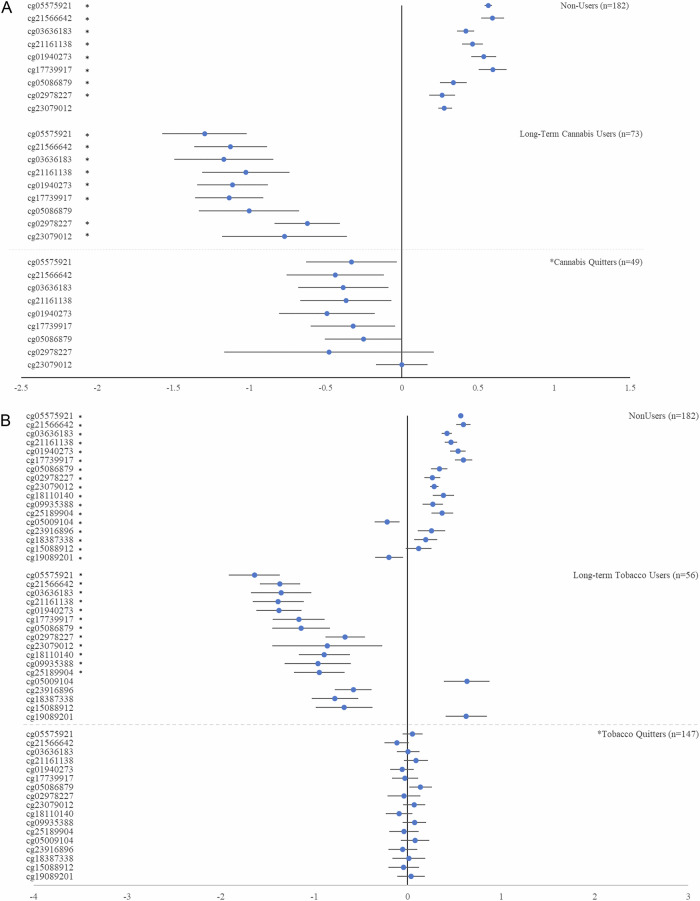
A comparison of cannabis and tobacco quitters with cannabis/tobacco non-users and long-term users. Panel **A** Cannabis quitters versus cannabis/tobacco non-users and long-term cannabis users. Panel **B** Tobacco quitters versus cannabis/tobacco non-users and long-term tobacco users. Means are crude means, standardized (M = 0.00, SD = 1.00) on the representative cohort. Therefore, means of 0.00 reflect the representative cohort norm. Error bars are 95% confidence intervals. Statistical tests used robust regression, with covariates (sex, principal components indexing technical variation, and white blood cell counts). Asterisks (*) indicate that the comparison with the quitter group was statistically significant at α adjusted for multiple testing (cannabis = 9 tests; tobacco = 17 tests).

Cannabis quitters showed a pattern of DNA methylation that was intermediate between long-term cannabis users and lifelong non-users (Fig. 3A, Table S7).

Likewise, tobacco quitters showed a pattern of DNA methylation that was intermediate between long-term tobacco users and lifelong non-users (Fig. 3B, Table S8).

Further, longer cannabis quit length was dose-dependently associated with increases in methylation at age 45, after adjusting for all covariates (Table S9). Specifically, whereas long-term cannabis users who had not quit showed hypomethylation at age 45, former cannabis users showed less extreme hypomethylation with each additional increment in quit length (i.e., longer quit length was positively associated with methylation).

## Discussion

This study characterized the DNA methylation profiles of long-term cannabis users in midlife, re-evaluating a set of 246 methylation sites shown in previous research to be associated with cannabis use. Key findings are as follows. First, 16 sites of cannabis-related differential DNA methylation were replicated across our comparisons of long-term cannabis-user versus non-user groups and our tests of dose-response associations, the latter of which used prospectively-assessed persistence of regular cannabis use from age 18–45 as the continuously-measured exposure. Second, when tests of dose-response associations were adjusted for a number of potential confounders (childhood socioeconomic deprivation; low childhood self-control; family history of substance dependence; and persistence of tobacco, alcohol, and illicit drug use), persistence of regular cannabis use remained robustly associated with hypomethylation of nine sites: cg05575921, cg21566642, cg03636183, cg21161138, cg01940273, cg17739917, cg05086879, cg02978227, cg23079012. Third, the nine sites were not specific to long-term cannabis users; long-term tobacco users showed hypomethylation of those same sites. Fourth, cannabis-related methylation sites showed biologically meaningful associations with gene expression. Fifth, the group of cannabis quitters showed less extreme DNA hypomethylation than long-term cannabis users, suggesting that cessation of cannabis use has the potential to reverse altered DNA methylation.

It is worth noting that most of the previously published markers of cannabis-associated differential DNA methylation were not replicated in this study. One factor might be our focus on cannabis use that is long-term, a difference from several prior studies that reported on less sustained use. However, our record can be compared to the literature where among the 246 previously published cannabis-related CpG sites, only one was reported by more than one study: cg05575921 [16, 20, 22] (Table S2). Another factor may be test-retest reliability. Our team previously documented the highly variable, but on average low, test-retest reliability of ~450,000 CpG sites assayed once using the 450 K BeadChip and once using the EPIC BeadChip, in the same DNA sample at the same time (mean intraclass correlation [ICC] = 0.21) [49]. Using that database (https://osf.io/83ucs/), we obtained the test-retest reliability of the 246 CpG sites that were the focus of the current study. Of the 246 sites, 156 had test-retest data because they were present on both the 450k and EPIC BeadChips, and their average ICC was only 0.28 (SD = 0.30, range = −0.13, 0.98). Sites that do not yield the same value if re-tested have limited utility. Importantly, the sites with better test-retest reliability showed the largest effect sizes in our tests of dose-response associations for persistence of regular cannabis use (r = 0.32, *p* < 0.0001) and persistence of tobacco dependence (r = 0.35, *p* < 0.0001). One possibility for future research, which may improve reproducibility, is to pre-screen for reliable CpG sites [49]. Another possibility is to use epigenetic measures that are enriched for reliable CpG sites [49]. For example, with one exception [50], several recent studies have shown that cannabis use is associated with accelerated epigenetic aging [51–53], using epigenetic clocks that correlate with physiological deterioration and a broad range of health indices.

Despite the relatively low replication rate, nine cannabis-related methylation sites were robust to adjustment for a range of covariates, and six of those sites with available gene expression data (cg05575921, cg21566642, cg03636183, cg21161138, cg01940273, cg23079012) were related to gene expression in a biologically meaningful way. Specifically, cannabis-related hypomethylation was associated with higher expression of the following genes: *AHRR, P2RY6, LRRN3, GPR15*, and *DSC2*. These genes are implicated in the metabolism of environmental toxins; immune system function and inflammatory response; and neurodevelopment, synaptogenesis, and neuroplasticity [54–58]. Higher expression is linked with some cancers (*AHRR, DSC2*) [59, 60], poorer prognosis of lung cancer (*P2RY6*) [55], and cardiovascular diseases (*GPR15, DCS2*) [61, 62]. Most of these genes have been implicated in tobacco smoking. Our findings are consistent with previous studies documenting that the cannabis-related methylation sites reported here are related to tobacco smoking and lung cancer (Table S10). However, unlike tobacco, cannabis use is not clearly associated with lung cancer [1] and is inconsistently associated with elevated risk of cardiovascular events [63].

The high degree of overlap between the methylation profiles of long-term cannabis users and long-term tobacco users has implications for the specific mechanisms underlying cannabis-related differential methylation. Although our findings showed that some associations between long-term cannabis use and DNA methylation were robust to adjustment for long-term tobacco use, it is difficult to disentangle cannabis effects from tobacco effects because most people who use cannabis also use tobacco. One possibility is that our analyses of long-term cannabis use did not fully account for tobacco effects, despite covariate adjustment, and tobacco use explains the cannabis-related differential methylation. However, another possibility is that cannabis and tobacco may independently affect DNA methylation. Importantly, one of the nine cannabis-associated sites from our study, cg05575921, has been shown to be associated specifically with tobacco smoking and not vaping or use of non-combustible tobacco products [8], which suggests smoking as a potential common mechanism. We found that long-term cannabis users and long-term tobacco users showed hypomethylation of cg05575921, but the effect was smaller for long-term cannabis users. The smaller effect for long-term cannabis users, and lack of a clear association between cannabis use and smoking-related health outcomes such as lung cancer, may be explained by the less intensive patterns of use typical of cannabis relative to tobacco. As cannabis use intensity increases [15], associations with lung cancer and other diseases could emerge.

A key question is whether THC exposure might also be a mechanism by which cannabis alters methylation. Rodent studies have shown that THC administration alters DNA methylation [64]. However, if THC were a mechanism underlying cannabis-related methylation in the present study, it is perhaps surprising that there were few methylation sites that were unique to cannabis. In this regard, it is worth noting that THC and nicotine affect shared brain pathways, including pathways that underlie reward, mood, and cognition. For example, despite binding to different brain receptors, THC and nicotine result in altered neurotransmission (e.g., increased dopamine release) [65], which could affect the epigenome in similar ways [66]. Moreover, the endocannabinoid and nicotinic systems appear to interact, with one system modulating the other [65]. Strikingly, epidemiological studies have documented that cannabis and tobacco relate similarly to a number of health outcomes, including psychosis [67], cognitive impairment [68], MRI-assessed brain structure [41], and periodontal disease [69]. This epidemiological evidence underscores the possibility that cannabis and tobacco use share biological pathways to health outcomes.

Quitting cannabis may at least partially reverse cannabis-related alterations to the epigenome. Cannabis quitters showed DNA methylation profiles that were intermediate between long-term cannabis users and non-users. Tobacco quitters showed the same pattern. The finding for tobacco has been shown before [70, 71] and is consistent with research documenting that tobacco cessation improves health outcomes [72]. The finding for cannabis is new and suggests that cannabis cessation may improve health outcomes.

This study has limitations. First, findings are based on a single New Zealand (NZ) cohort born in the 1970s when cannabis was much less potent (i.e., characterized by lower THC concentrations) than it is today [73, 74]. Relatedly, the heaviest cannabis users in the Dunedin cohort had used cannabis on an almost daily basis for years, but they comprised only ~6% of the cohort. Recent data from the US show that the number of daily cannabis users is increasing [15]. If long-term frequent use of high-potency cannabis underlies cannabis effects on methylation, then our study may have underestimated cannabis-methylation associations. In terms of how NZ currently compares with the US and Europe, medical cannabis is legal in NZ but non-medical use (i.e., recreational use) is not, similar to many American states. The NZ medical cannabis market has shifted toward high-THC products, and the potency of medical and illegal market cannabis products in NZ is similar to the US [75]. The rate of disability adjusted life years attributable to cannabis use disorder is higher in NZ than in the US and UK [76].

Second, the DNA is from blood, and research is needed to determine if findings replicate in other tissues (e.g., lung, which is obtained much more invasively than blood). Third, the study cannot address the mechanisms of cannabis-associated hypomethylation. Most people who use cannabis smoke it [4, 5], and although smoking is one plausible mechanism, research is needed to ascertain whether users who primarily vape or ingest cannabis also show differential DNA methylation.

Fourth, this paper focused on cannabis use. We included tobacco comparisons because tobacco associations provide important context for interpreting cannabis associations. However, there are tobacco-associated DNA methylation markers that we do not report on here, because we selected previously published cannabis-associated methylation markers for replication. Nonetheless, all 17 tobacco-associated markers of differential DNA methylation that replicated across tests of group comparisons and tests of dose-response associations are reported as tobacco-associated in the EWAS catalog in the same direction as we report here. Fifth, in group comparisons, small N’s may have reduced statistical power (*n* = 49–182). However, our dose-response tests had ample power (*n* = 787). Finally, this study is observational and cannot determine causality. Nonetheless, we found evidence of dose-response associations, which bolsters biological plausibility of a causal effect. Moreover, dose-response tests addressed confounding by adjusting for factors known to characterize long-term cannabis users: socioeconomically deprived circumstances, poor childhood self-control, family history of substance dependence, and persistent tobacco, alcohol, and illicit drug use. The field generally lacks tests of dose-response associations. Our findings represent a unique contribution.

This study has several implications. First, long-term cannabis use could affect DNA methylation in similar ways to tobacco, possibly at least partly though the shared mechanism of smoke inhalation. Second, research is needed to ascertain whether cannabis-related hypomethylation and associated alterations in gene expression result in the development of disease. Third, at a time when cannabis and cannabinoids are increasingly touted and used for salutary effects, it is important to recognize that the epigenome may be responsive to long-term cannabis use regardless of whether use is for medical or non-medical reasons. Finally, cannabis cessation could reverse altered DNA methylation.

## Supplementary information

Supplement

## Data Availability

Dunedin Study data is available via managed access at https://dunedinstudy.otago.ac.nz/for-investigators/policy-statement-code-of-practice.

## Code availability

All scripts used in the analyses are available here: https://github.com/MoffittCaspiLab/Meier_MolecularPsychiatry_2025.
