# Correction: DNA methylation profiles of long-term cannabis users in midlife: a comprehensive evaluation of published cannabis-associated methylation markers in a representative cohort

**DOI:** 10.1038/s41380-025-03107-9

**Published:** 2025-07-04

**Authors:** Madeline H. Meier, Karen Sugden, Terrie E. Moffitt, Benjamin S. Williams, Kyle J. Bourassa, Renate Houts, Sandhya Ramrakha, Reremoana Theodore, Avshalom Caspi

**Affiliations:** 1 Department of Psychology, Arizona State University, Tempe, AZ USA; 2 Department of Psychology & Neuroscience, Duke University, Durham, NC USA; 3 Department of Psychiatry and Behavioral Sciences, Duke University School of Medicine, Durham, NC USA; 4 Center for Genomic and Computational Biology, Duke University, Durham, NC USA; 5 Social, Genetic and Developmental Psychiatry Centre, Institute of Psychiatry, Psychology and Neuroscience, King’s College London, London, England; 6 PROMENTA Research Center, University of Oslo, Oslo, Norway; 7 Mid-Atlantic Mental Illness Research, Education and Clinical Center, Durham VA Health Care System, Durham, NC USA; 8 Department of Psychology, Georgetown University, Washington, DC USA; 9 Center for the Study of Aging and Human Development, Duke University Medical Center, Durham, NC USA; 10 Dunedin Multidisciplinary Health and Development Research Unit, Department of Psychology, University of Otago, Dunedin, New Zealand

**Keywords:** Genetics, Molecular biology

Correction to: *Molecular Psychiatry* 10.1038/s41380-025-03042-9, published online 27 June 2025

In this article, the order in which the authors appeared in the author list was incorrectly given as Avshalom Caspi, Karen Sugden, Terrie E. Moffitt, Benjamin S. Williams, Kyle J. Bourassa, Renate Houts, Sandhya Ramrakha, Reremoana Theodore and Madeline H. Meier where it should have been Madeline H. Meier, Karen Sugden, Terrie E. Moffitt, Benjamin S. Williams, Kyle J. Bourassa, Renate Houts, Sandhya Ramrakha, Reremoana Theodore and Avshalom Caspi. The original article has been corrected.

