# Supplementary material for: DNA methylation profiles of long-term cannabis users in midlife: a comprehensive evaluation of published cannabis-associated methylation markers in a representative cohort
Source: Mol Psychiatry. 2025 Jun 27;30(10):4576–90. doi: 10.1038/s41380-025-03042-9 (PMC12436150; doi:10.1038/s41380-025-03042-9)
Supplement: Supplementary file 1 — Supplement [file 41380_2025_3042_MOESM1_ESM.pdf]

Supplemental Text. Methods for tests of associations between DNA methylation and gene expression levels.

Expression data were generated from whole-blood RNA using the Affymetrix PrimeView Human Gene Chip (Affymetrix, CA, USA). Briefly, these arrays simultaneously interrogate more than 38,000 gene transcripts across the entire genome. Whole-blood RNA samples collected via PaxGene Blood RNA tubes (Qiagen, CA, USA) at age 38 were assayed. Samples were arranged into batches of 60. Array analysis was performed by the Duke University Genomic and Computational Biology Microarray Core Facility using the Affymetrix GeneChip system (Affymetrix). Prior to hybridization, Total RNA were assessed for quality with Agilent 2100 Bioanalyzer G2939A (Agilent Technologies, Santa Clara, CA)) and Nanodrop 8000 spectrophotometer (Thermo Scientific/Nanodrop, Wilmington, DE). Samples with RIN ≥ 6 were then subject to globin mRNA depletion using the GLOBINClear –human kit (Ambion, Thermo Fisher Scientific, MA, USA). RNA samples from 843 individuals were assayed. Data quality control and RMA normalization was carried out using the '*affy'* Bioconductor package in the R statistical programming environment. Samples from 836 age-38 participants passed our QC pipeline.

To permit control for technical variation, we used the following microarray-based quality metrics to residualize probeset values following the approach described by Peters *et al*.[1]: mean of positive match probesets, mean of positive control probesets, standard deviation of positive control probesets, mean of negative control probesets, standard deviation of negative control probesets, mean of all probesets, standard deviation of all probesets, and relative log expression mean of all probesets, along with sex, array batch, and RIN. To control for cell type composition, we also included white cell-type counts measured using flow cytometry (Sysmex Corporation, Japan) in whole blood samples taken concurrently with the RNA sample.

Genome-wide quantification of DNA methylation for gene expression correlation analyses.

Because gene expression was measured when study members were 38 years old, for the correlational analyses we employed additional DNA methylation data that was collected concurrently at age 38. Whole blood was collected in 10mL K_2EDTA tubes from 90% (N=857) of participants at age 38. DNA was extracted from the buffy coat using standard procedures. Study members who did not provide blood provided buccal swabs, but these were not included in our methylation analysis to avoid tissue-source confounds.

We assayed 835 blood samples (out of 857); 22 samples were not useable. ~500ng of DNA from each sample was treated with sodium bisulfite using the EZ-96 DNA Methylation kit (Zymo Research, CA, USA). DNA methylation was quantified using the Illumina Infinium HumanMethylation450 BeadChip ("Illumina 450K array") run on an Illumina iScan System (Illumina, CA, USA) at the Molecular Genomics Core at the Duke Molecular Physiology Institute.

Data were processed and normalized using the '*methylumi'* (v2.14.0) Bioconductor package from the R statistical programming environment and subjected to quality control (QC) analyses. Samples were removed if the average detection p-value was >= 0.001. To confirm genetic identity of the DNA samples, we assessed genotype concordance between SNP probes on the 450K array and data generated using Illumina OmniExpress12v1.1 genotyping BeadChips. Principal components analysis was performed on the full, normalized dataset and the first two components plot. Samples formed two major clusters separating on the first component, which corresponded to recorded sex. This was used to confirm sex assignment. Samples from 819 age-38 participants passed our QC pipeline.

To permit control for technical variation, we used residualized DNA methylation values for the principal components described above. To control for cell type composition, we also included white cell-type counts measured using flow cytometry (Sysmex Corporation, Japan) in whole blood samples taken concurrently with the DNA sample.

DNA methylation- Gene expression correlation analysis.

To test whether there were any potentially meaningful biological relationships between the DNA methylation probes associated with cannabis use and gene expression, we examined correlations between methylation probe levels and whole-genome gene expression (N=45,374 probesets). Both DNA methylation and gene expression data were available for 808 study members at age 38. Because DNA methylation data were assayed using 450K BeadChip arrays at age 38, not all identified probes were available. Of the nine cannabis-related probes that replicated in tests of group comparisons and tests of dose-response associations at age 45 and were robust to adjustment for all covariates), six were present in the data at age 38: cg05575921, cg21566642, cg03636183, cg21161138, cg01940273, cg23079012. All six probes were statistically significantly associated with long-term cannabis use at age 38, like at age 45 (**Table S6**). (The criteria for long-term cannabis users at age 38 were analogous to the criteria at age 45 – i.e., used cannabis weekly or were dependent at age 38 and had used cannabis weekly or more frequently at one or more previous study waves. There were 76 and 74 study members in the long-term cannabis use groups at age 38 and age 45, respectively. Overlap between the age-38 and age-45 long-term cannabis use groups was substantial (kappa=0.68).)

We used Spearman's *rho* and Bonferroni-corrected p-value cutoffs ($p = 1.02x10^{-6}$) to identify significant associations between DNA methylation levels and gene expression. We further filtered significant associations to only those where rho was negative; this is to reflect the expected direction of association between DNA methylation and gene expression levels if direct regulation of gene expression through DNA methylation were to exist.

All six DNA methylation probes tested were significantly associated with expression levels of at least one probeset. Two of these were cis associations (i.e., occurred within the same region of DNA); these probes were at the *AHRR* locus (cg05575921 and cg21161138) and were associated with *AHRR* gene expression. Many of the genes correlated with DNA methylation have been previously implicated in smoking and smoking-related behavior (e.g. *LRRN3, AHHR, GPR15*). To visualize these relationships, we plotted circos plots for the results of each DNA methylation probe-gene expression test using the '*circlize'* R package (v0.4.16). These associations are shown in **Figure S3**.

Supplemental Text References

1. Peters MJ, et al. The transcriptional landscape of age in human peripheral blood. *Nat Commun*. 2015;**6**:8570.
2. Bowtell DD. Rapid isolation of eukaryotic DNA. *Anal Biochem*. 1987;**162**(2):463-5.
3. Jeanpierre M. A rapid method for the purification of DNA from blood. *Nucleic Acids Res*. 1987;**15**(22):9611.

Table S1. Published studies of cannabis-related DNA methylation. (Studies are included if they reported on specific CpG sites and were published by August 2024. They are ordered by publication year.)					
Study	Sample	Exposure	Outcome	Covariates	Results
Osborne et al., 2020 (16)	The Christchurch Health and Development Study – a longitudinal study that followed participants from birth to age 40. Analyses were based on the cohort followed to approximately age 30 (N=987), from which 96 participants for whom a blood sample was available were selected.	Regular cannabis users (n=48) were matched with non-users (n=48), based on sex (n=37 male, n=11 female).<br><br>Regular cannabis users were split into two subsets – a subset of 24 who never used tobacco and a subset of 24 who used tobacco. Non-users had never used cannabis or tobacco.<br><br>Regular cannabis users had diagnosed with cannabis dependence, or had used cannabis on daily basis for at least three years, by age 28. N=6 had quit using cannabis by age 28 but still met diagnostic criteria for cannabis dependence.<br><br>All regular cannabis users had smoked it. Mean duration of use was 9 years.	DNA methylation from peripheral whole blood taken at age 28. EPIC BeadChip. N=700,296 CpG sites (EWAS).	Sex, socioeconomic status, batch, population stratification, cell type. Analyses were stratified by cannabis users who had and had not used tobacco.	Cannabis+tobacco users showed hypomethylation of 5 CpG sites, after correcting for multiple testing: cg05575921, cg21566642, cg03636183, cg01940273, cg17739917.<br><br>Cannabis-only users did not show differential methylation after correcting for multiple testing.<br><br>Pathway enrichment analysis for cannabis-only group for CpG sites with p<.001: The hypermethylated CpG sites (n=420) showed enrichment in the arrhythmogenic right ventricular cardiomyopathy, long-term potentiation, cAMP signaling, adrenergic signaling in cardiomyocytes, glutamatergic synapse, hypertrophic cardiomyopathy, dilated cardiomyopathy, and nicotine addiction pathways at an adjusted p<.05. The hypomethylated sites (n=101) showed no significant KEGG pathways after correction for multiple testing. When considering all differentially methylated CpG sites (hyper and hypomethylated) at p<.001, there was enrichment for genes involved in the glutamatergic synapse, arrhythmogenic right ventricular cardiomyopathy, and long-term potentiation pathways.<br><br>The 5 significant CpGs for cannabis+tobacco users are included in our replication set (shown in Table S2)

Table S1. Published studies of cannabis-related DNA methylation. (Studies are included if they reported on specific CpG sites and were published by August 2024. They are ordered by publication year.)					
Study	Sample	Exposure	Outcome	Covariates	Results
					and have been shown in previous studies to be associated with tobacco use.
Markunas et al., 2021 (17)	The Sister Study – a longitudinal study of 50,884 women ages 35-75 at risk for breast cancer ascertained across the US from 2003-09. The analytic sample was from a case-cohort sub-study of 2,878 non-Hispanic white women who were breast-cancer free at the time of the blood draw. Following exclusions, the final analytic sample was 2,583 women. Discovery N=1,730. Replication N=853.	Self-reported ever vs. never cannabis use. Ever use: n=855 in Discovery sample; n=392 in Replication sample.<br><br>Follow-up analyses in the combined sample considered total duration of use divided into quartiles (upper≥5 years, lower≤1 year) and age at initiation (mean age=21 years).	DNA methylation from peripheral whole blood taken at mean age ~57. 450K BeadChip. N=428,072 CpG sites (EWAS).	Age, incident breast cancer status, tobacco smoking, alcohol use, laboratory plate, DNA extraction method, technical artifacts, and blood-cell-type proportions. Sensitivity analyses considered other potential confounders: perceived stress, family income while growing up,	Lifetime cannabis use was associated with one CpG at a false discovery rate < 0.10: cg15973234. This CpG site was hypomethylated. Further adjustment for perceived stress, family income, body mass index, and depression did not alter results. This finding replicated in the Replication sample and in the combined sample. However, cg15973234 was unrelated to total duration of cannabis use and age of initiation of use.<br><br>In an analysis using the top 62 most significant CpGs from the Discovery Sample ($p < 1 \times 10^{-4}$), LASSO regression resulted in a 50-CpG classifier of lifetime cannabis use, with

Table S1. Published studies of cannabis-related DNA methylation. (Studies are included if they reported on specific CpG sites and were published by August 2024. They are ordered by publication year.)					
Study	Sample	Exposure	Outcome	Covariates	Results
				body mass index, history of depression. Further, for significant EWAS findings, analyses in the combined sample were stratified by subsequent breast cancer, alcohol, and tobacco.	an AUC of 0.74 in the Discovery sample and 0.54 in the Replication sample. 48 of these 50 CpGs are included in our replication set (shown in Table S2). Two were excluded for not being available on the Dunedin EPIC 850K BeadChip.
Clark et al., 2021 (18)	Great Smoky Mountain Study – a longitudinal study of 1,420 children ages 9-13 recruited from rural counties in the southeast US beginning in 1993. Analyses were based on 525 participants ages 9-21 who had a blood spot taken.	Cannabis use disorder (CUD) symptom count, obtained from structured diagnostic interviews during the same visit the blood spots were taken.<br><br>N=42 participants with ≥1 symptom of CUD. Mean age of this group=17.1 (SD=2.30).	DNA methylation from peripheral whole blood taken in adolescents. Illumina NextSeq 500 System was used for the EWAS.	Technical artifacts, cell-type proportions, age, age squared, race, sex, regular cigarette use.	Cannabis use disorder symptoms were associated with 45 CpGs at a false discovery rate of 0.10. Pathway analysis showed 19 pathways, including those related to cholinergic synapse and serotonergic synapse.<br><br>None of these CpGs are included in our replication set (Table S2) because none are available on the Dunedin EPIC 850K BeadChip
Wiedmann et al., 2022 (19)	Adolescent psychiatric outpatients with chronic cannabis use (n=9) and comparison individuals with no lifetime illicit substance use (n=9), matched on age, sex, and psychiatric disorders.	Chronic cannabis users were compared with non-users. Chronic use was defined as at least weekly use during the past year and cannabis-related problems. Chronic users had used cannabis for an average of 21 days per month in the past year. Of the 9 chronic cannabis users, 6 met criteria for ICD-10 cannabis dependence, and 3 met criteria for ICD-10 harmful use.	DNA methylation from peripheral whole blood taken at mean age ~15.5. EPIC BeadChip. N=866,238 CpG sites. (EWAS).	Groups were matched for age, sex, and psychiatric disorders. Tobacco and alcohol use frequency were covariates.	Principal component analysis was used to compare groups on whole genome methylation. Correlations were used to test associations between extent of cannabis use and CpGs.<br><br>There was a large effect difference between groups on whole genome DNA methylation profiles, but the difference was non-significant.<br><br>In correlational analyses, extent of cannabis use was associated with 6 CpGs, after adjusting for multiple testing: cg17285328, cg20777378, cg04904300, cg08923376,

Table S1. Published studies of cannabis-related DNA methylation. (Studies are included if they reported on specific CpG sites and were published by August 2024. They are ordered by publication year.)					
Study	Sample	Exposure	Outcome	Covariates	Results
		Extent of cannabis use was defined as the average quantity of use per day in the past year (in grams) multiplied by the frequency of days of use in the past year.			cg04270414, cg23767840. All sites were hypomethylated. These 6 CpGs are included in our replication set (Table S2).
Nannini et al., 2023 (20)	Coronary Artery Risk Development in Young Adults Study – a study of 5,115 Black and White individuals ages 18-30 recruited from the US in 1985-86. Participants were followed for over 35 years. Analyses report on DNA methylation at follow-up years 15 and 20 for a randomly selected subset of 1,200 participants with available whole blood.<br><br>At Year 15: n=1,023<br>At Year 20: n=883	Recent cannabis use at follow-up years 15 (mean age 40) and 20 (mean age 45) was defined as the number of days of cannabis use in the past 30 days and was based on self-reports. At year 15: n=140 with recent use, n=883 with no recent use. At year 20: n=113 with recent use, n=770 with no recent use.<br><br>Cumulative use from baseline to follow-up years 15 and 20 was calculated based on past-month reports of number of days of cannabis use. Past-month cannabis use was assumed to be representative of cannabis use between assessments, and total number of days of use from baseline to year 15 and year 20 were summed and divided by 365 to yield a measure of	DNA methylation from peripheral whole blood taken at years 15 and 20. EPIC BeadChip. N=841,639 CpG sites (EWAS).	Technical bias, leukocyte cell-type population, age, sex, race, study center, education, tobacco smoking status, physical activity, alcohol use.	**Cross-sectional associations:**<br>At year 15, recent and cumulative cannabis use were associated with 22 and 31 CpG sites at a false-discovery rate p<.05, respectively. (N=53 total CpG sites.)<br><br>At year 20, recent and cumulative cannabis use were associated with 132 and 16 CpG sites at a false-discovery rate p<.05, respectively. (N=148 total CpG sites.)<br><br>One CpG site in common across all four analyses: cg05575921.<br><br>**Longitudinal associations:**<br>Of the 22 and 132 CpG sites associated with *recent cannabis use* at years 15 and 20, 13 and 124 were associated with longitudinal change in a consistent direction. Of the 31 and 16 CpG sites associated with *cumulative cannabis use* at years 15 and 20, 20 and 16 were associated with longitudinal change in a consistent direction.<br><br>**Stratified analyses:**<br>In cross-sectional analyses stratified by sex, race, and tobacco smoking status, regression coefficients were highly

Table S1. Published studies of cannabis-related DNA methylation. (Studies are included if they reported on specific CpG sites and were published by August 2024. They are ordered by publication year.)					
Study	Sample	Exposure	Outcome	Covariates	Results
		"cannabis-years," with one cannabis-year representing daily cannabis use for one year. Cannabis years ranged from a mean of ~5 to 6 years among recent cannabis users at years 15 and 20 and 0.4 and 0.5 years among participants who did not use cannabis in the past month at years 15 and 20.			correlated for men and women, for White and Black participants, and for cannabis users who were and were not tobacco smokers.<br><br>**Replication analyses:**<br>In replication analyses of 31 cannabis-associated CpGs reported in previous studies, between 6 and 8 CpG sites showed replicated associations with recent or cumulative cannabis use at years 15 and 20 after Bonferroni correction.<br><br>**Pathway analyses:**<br>In pathway analyses at year 15, the top pathways associated with recent marijuana use were related to MAPK signaling, diseases of signal transduction, and the neuronal system; the top pathways associated with cumulative use included Rho GTPase, cell proliferation and apoptosis, and depolarization. At year 20, the top pathways associated with recent marijuana use were related to dopamine synapses, diseases of signal transduction, transcription, human papillomavirus infection, and oxytocin signaling; the top pathways associated with cumulative use included diseases of signal transduction, transcription regulation by RUNX2, WNT signaling, human papillomavirus infection, and oxytocin signaling.<br><br>Overall, the study identified 182 unique cannabis-associated CpG sites at false-

Table S1. Published studies of cannabis-related DNA methylation. (Studies are included if they reported on specific CpG sites and were published by August 2024. They are ordered by publication year.)					
Study	Sample	Exposure	Outcome	Covariates	Results
					discovery rate p<.05. Of these, 180 were available on the Dunedin EPIC 850K BeadChip and were included in our replication set (Table S2).
Carreras-Gallo et al., 2023 (21)	The study sample was drawn from the TruDiagnostic DNA biobank – a predominantly US-based cohort of 13,108 individuals. The analytic sample was 3,424 participants ages 13-97, recruited in calendar years 2020-22, with mean age 52.9 years.	Self-reported cannabis use: never (n=2,908, 88.8%), special occasions (n=180, 5.5%), once a week (n=46, 1.4%), 3-5 times a week (n=73, 2.2%), regularly (n=68, 2.1%).	DNA methylation taken from peripheral whole blood. EPIC BeadChip. N=745,150 CpGs (EWAS).<br><br>Epigenetic clocks: Horvath multi-tissue, Hannum, PhenoAge, GrimAge, DunedinPACE, telomere length.	Slide, cell type, surrogate variables to remove batch effects, sex, age, ethnicity, body mass index, education, alcohol use, tobacco use.	Frequency of cannabis use was not associated with any CpG site when adjusting for multiple testing. However, frequency of cannabis use was associated with 195 CpGs at a p-value suggested by the EWAS catalog ($p<1*10^{-4}$). Pathway analysis for these 195 CpGs found an enrichment of myelin assembly. Cannabis use was not associated with any epigenetic clock.<br><br>Since no CpGs survived adjustment for multiple testing, none were included in our replication set (Table S2).
Garrett et al., 2024 (22)	Participants were drawn from the multi-site Veterans Affairs Mid-Atlantic Mental Illness Research, Education and Clinical Center Study of Post-Deployment Mental Health, a cohort of Iraq/Afghanistan-era veterans enriched for posttraumatic stress disorder (n=2,310 participants; n=1,109 non-Hispanic Black, n=1,201 non-Hispanic White).<br>Main findings were meta-analyzed across four analysis groups, stratified by race and BeadChip, with sex and post-traumatic stress disorder (PTSD)	Lifetime cannabis use disorder (CUD) obtained via structured diagnostic interview. 11% of the sample had a lifetime diagnosis of CUD (n=249). The prevalence of CUD was the same for Black and White participants.<br><br>Post-hoc analyses examined cumulative cannabis use disorder (never: n=1,957; former: n=215; current: n=34) in analyses of CpG sites associated with lifetime CUD.	DNA methylation taken from peripheral whole blood at mean age 37 years. Either the 450K BeadChip or the EPIC BeadChip was used. N=423,945 CpG sites (EWAS).	Associations between CUD and CpGs were tested in each analysis group separately, with analysis group stratified by race and BeadChip, and sex and post-traumatic stress disorder (PTSD) randomized. Experimental batch and tobacco smoking status were included as covariates. Unobserved confounders, such as cell type	Lifetime CUD was associated with four CpGs after controlling for current tobacco smoking and correcting for multiple testing: cg05575921, cg23079012, cg22112841, cg08760398. All CpGs were hypomethylated except cg22112841, which was hypermethylated. In a sensitivity analysis among never-smokers of tobacco (conducted in sample of Black participants only due to the distribution of CUD and tobacco smoking in each analysis group), two CpGs were associated with lifetime CUD after correction for multiple testing: cg05575921 and cg24405700. Post-hoc analyses of cumulative CUD for the 4 significant CpGs were similar to results for lifetime CUD.

Table S1. Published studies of cannabis-related DNA methylation. (Studies are included if they reported on specific CpG sites and were published by August 2024. They are ordered by publication year.)					
Study	Sample	Exposure	Outcome	Covariates	Results
	randomized: White 450K (n=176), White EPIC (n=1,025), Black 450K (n=274), Black EPIC (n=835).			composition, were taken into account using random effect components. Results were then meta-analyzed.<br><br>Post-hoc analyses of cumulative cannabis use disorder, which used the full sample, included age, sex, race, BeadChip, cell type estimates, and current smoking status as covariates.	All 5 significant CpGs (cg05575921, cg23079012, cg22112841, cg08760398, cg24405700), were included in our replication set (Table S2).
Fang et al., 2024(23)	A meta-analysis of participants from seven cohorts: the Sister Study, Gulf Long-term Follow-up Study, Netherlands Twin Register, Veteran Aging Cohort Study, Finnish Twin Cohort, Avon Longitudinal Study of Parents and Children, and UK Adult Twin Registry. The analytic sample comprised 9,436 participants (n=4,190 ever cannabis users, n=5,246 never users).	Lifetime cannabis use based on self- or parent-report (n=4,190 ever users, n=5,246 never users).	DNA methylation taken from peripheral whole blood at mean ages ranging from 17 to 59 years. Either the 450K BeadChip or the EPIC BeadChip. N=452,453 CpG probes available on both the 450K and EPIC BeadChips.	Technical covariates, blood cell type estimation, sex, age, and, in additional analyses, cigarette smoking (current, former, never).	The EWAS meta-analysis found that lifetime cannabis use was associated with 608 CpGs at the false discovery rate threshold of 5%, 500 of which were previously shown to be associated with cigarette smoking. When cigarette smoking was included as a covariate, lifetime cannabis use associated with four CpGs: cg01101459, cg22572071, cg15280538, and cg00813162. None of these had been reported significant in previous EWAS of tobacco smoking after accounting for multiple testing.<br><br>In analyses stratified by ancestry, effect sizes were highly correlated across European American and African American groups. In analyses stratified

Table S1. Published studies of cannabis-related DNA methylation. (Studies are included if they reported on specific CpG sites and were published by August 2024. They are ordered by publication year.)					
Study	Sample	Exposure	Outcome	Covariates	Results
					by sex, effect sizes were highly correlated across males and females.<br><br>In an EWAS meta-analysis of participants who never smoked cigarettes (N=4,146), lifetime cannabis use was associated with one CpG at the false discovery rate threshold of 5%: cg14237301.<br><br>In replication analyses of 7 nominally significant CpGs from Markunas et al., 30 significant CpGs from Osborne et al., and 40 significant CpGs from Nannini et al., 7 (100%), 10 (33%), and 4 (10%) replicated, after Bonferroni adjustment.<br><br>A methylation score was created based on the top 50 CpGs from an EWAS meta-analysis without the Sister Study-the largest cohort. The methylation score explained 3.79% of the variance in lifetime cannabis use in the Sister Study (not adjusted for tobacco smoking) and .58% of the variance (adjusted for tobacco smoking). Among never-smokers, it explained .91% of the variance.<br><br>Effect sizes from analyses adjusting for tobacco use and additionally adjusting for alcohol use and body mass index were correlated 0.99 with effect sizes from analyses not adjusting for alcohol and body mass index, using the top CpG sites.

Table S1. Published studies of cannabis-related DNA methylation. (Studies are included if they reported on specific CpG sites and were published by August 2024. They are ordered by publication year.)					
Study	Sample	Exposure	Outcome	Covariates	Results
					Of the five CpGs that were epigenome-wide significantly associated with lifetime cannabis use (four from the meta-analysis of all participants, adjusting for tobacco smoking [cg01101459, cg22572071, cg15280538, and cg00813162], and one from participants who never smoked tobacco [cg14237301]), 3 were significantly correlated with nearby gene expression in the brain (cg01101459, cg00813162, cg14237301), using data from the EWAS atlas for gene expression.<br><br>Enrichment analyses for the top CpGs from the meta-analysis adjusted for tobacco smoking showed enriched traits, including Crohn's disease, alcohol consumption, body mass index, and multiple sclerosis. Cannabis-associated CpGs in participants who never smoked tobacco showed overlap with CpGs previously associated with smoking, smoking cessation, lung function, and lung carcinoma.<br><br>Overall, the study identified 5 CpGs associated with lifetime cannabis use, after adjusting for multiple testing and taking into account tobacco smoking cg01101459, cg22572071, cg15280538, cg00813162, and cg14237301. All but one (cg15280538) were included in our replication set (Table S2). The excluded CpG was not available the Dunedin EPIC 850K BeadChip.
Note. EWAS=Epigenome wide association study. KEGG=Kyoto Encyclopedia of Genes and Genomes. ICD=International Classification of Diseases.					

Table S2. Cannabis-associated CpG sites identified in previous studies (n=246). (The 9 cannabis-associated CpG sites that replicated across our tests of group comparisons and our fully covariate-adjusted tests of dose-response associations are shaded in gray.)								
	CpG Site	Study	BeadChip	Genomic Location	Nearest Gene	Location Relative to Nearest Gene	Mean Age-45 Dunedin β	Test-Retest Reliability ICC (450k-EPIC)[a]
1	cg00250546	Nannini	EPIC	Chr5:22,168,242-22,168,242	CDH12	5'UTR	0.76	-
2	cg00500007	Nannini	EPIC	Chr13:35,624,620-35,624,620	NBEA	Body	0.71	-
3	cg00761236	Nannini	EPIC	Chr13:107,305,783-107,305,783	LINC00551	TSS	0.91	-
4	cg00785657	Nannini	EPIC	Chr19:35,168,593-35,168,593	ZNF302	1stExon	0.01	-
5	cg00813162	Fang	450K/EPIC	Chr14:69,443,313-69,443,363	ACTN1	Intron	0.63	0.70
6	cg01035812	Markunas	450K	Chr17:4,843,536-4,843,586	SLC25A11	TSS	0.03	-0.07
7	cg01039752	Markunas	450K	Chr16:81,439,587-81,439,637	GAN	Intergenic	0.88	0.32
8	cg01101459	Fang	450K/EPIC	Chr1:234,871,476-234,871,526	LINC01132	Intergenic	0.87	0.51
9	cg01198887	Markunas	450K	Chr6:166,907,834-166,907,884	RPS6KA2	Intron	0.95	0.14
10	cg01212491	Nannini	EPIC	Chr10:75,587,308-75,587,308	CAMK2G	Body	0.87	-
11	cg01372788	Nannini	EPIC	Chr12:124,122,424-124,122,424	GTF2H3	5'UTR	0.96	-
12	cg01443684	Nannini	EPIC	Chr1:39,314,427-39,314,427	RRAGC	Body	0.79	-
13	cg01472075	Markunas	450K	Chr16:69,984,926-69,984,976	CLEC18A	5'UTR	0.79	0.19
14	cg01668099	Nannini	EPIC	Chr11:130,026,797-130,026,847	ST14	Intergenic	0.80	0.54
15	cg01738710	Nannini	EPIC	Chr1:44,182,062-44,182,062	ST3GAL3	5'UTR	0.91	-
16	cg01806956	Markunas	450K	Chr1:9,460,781-9,460,831	SPSB1	Intergenic	0.34	0.56
17	cg01940273	Osborne	EPIC	Chr2:233,284,885-233,284,935	AC068134.5	Intergenic	0.72	0.73
18	cg01947805	Nannini	EPIC	Chr11:10,906,230-10,906,230	ZBED5-AS1	3'UTR	0.87	-
19	cg02038919	Nannini	EPIC	Chr7:43,232,885-43,232,885	HECW1	5'UTR	0.85	-
20	cg02235741	Markunas	450K	Chr13:99,853,131-99,853,181	UBAC2	TSS	0.02	0.04
21	cg02337960	Nannini	EPIC	Chr19:17,378,596-17,378,646	BABAM1	Intron	0.07	0.07
22	cg02473540	Markunas	450K	Chr19:58,570,453-58,570,503	ZNF135	Intergenic	0.25	0.76
23	cg02616769	Nannini	EPIC	Chr3:22,348,098-22,348,148	ZNF385D	Intron	0.91	0.32

Table S2. Cannabis-associated CpG sites identified in previous studies (n=246). (The 9 cannabis-associated CpG sites that replicated across our tests of group comparisons and our fully covariate-adjusted tests of dose-response associations are shaded in gray.)								
	CpG Site	Study	BeadChip	Genomic Location	Nearest Gene	Location Relative to Nearest Gene	Mean Age-45 Dunedin β	Test-Retest Reliability ICC (450k-EPIC)[a]
24	cg02646643	Nannini	EPIC	Chr3:184,026,702-184,026,752	PSMD2	3'UTR	0.67	0.00
25	cg02669268	Nannini	EPIC	Chr2:209,130,300-209,130,300	PIKFYVE	TSS	0.05	-
26	cg02703675	Nannini	EPIC	Chr11:12,159,354-12,159,354	MICAL2	TSS	0.49	-
27	cg02705374	Markunas	450K	Chr12:97,301,582-97,301,632	NEDD1	5'UTR	0.04	0.01
28	cg02905178	Nannini	EPIC	Chr4:7,025,903-7,025,953	TBC1D14	Intron	0.93	0.19
29	cg02978227	Nannini	EPIC	Chr3:98,292,027-98,292,027	ENSG00000285635	Intron	0.93	-
30	cg03093806	Nannini	EPIC	Chr15:40,331,376-40,331,426	SRP14	TSS	0.06	-0.02
31	cg03116409	Nannini	EPIC	Chr12:66,042,505-66,042,555	RP11-221N13.4	Intron	0.95	-0.06
32	cg03221819	Nannini	EPIC	Chr10:75,647,445-75,647,445	CAMK2G	Intergenic	0.94	-
33	cg03457142	Markunas	450K	Chr3:71,804,858-71,804,908	GPR27	3'UTR	0.59	0.92
34	cg03636183	Osborne	EPIC	Chr19:17,000,536-17,000,586	F2RL3	Coding region	0.76	0.63
35	cg03765885	Markunas	450K	Chr2:119,571,673-119,571,723	RP11-19E11.1	Intergenic	0.73	0.69
36	cg03802952	Nannini	EPIC	Chr2:45,411,645-45,411,645	LINC01121	Body	0.87	-
37	cg03905975	Nannini	EPIC	Chr8:128,637,338-128,637,338	ENSG00000286266	Intron	0.95	-
38	cg03991223	Nannini	EPIC	Chr1:55,542,588-55,542,588	USP24	Body	0.96	-
39	cg04195527	Markunas	450K	Chr2:118,846,240-118,846,290	INSIG2	Exon	0.08	-0.05
40	cg04270414	Wiedman	EPIC	Chr11:64,807,234-64,807,284	RP11-399J13.3	3'UTR	0.90	0.94
41	cg04685163	Markunas	450K	Chr8:1,645,454-1,645,504	DLGAP2	Coding region	0.94	0.01
42	cg04711836	Nannini	EPIC	Chr17:39,882,259-39,882,259	HAP1	Body	0.89	-
43	cg04742550	Nannini	EPIC	Chr16:31,366,380-31,366,430	ITGAX	Intergenic	0.10	0.75
44	cg04831510	Nannini	EPIC	Chr6:129,250,695-129,250,745	MESTP1	Exon	0.95	0.01
45	cg04864586	Nannini	EPIC	Chr4:99,853,050-99,853,100	RP11-571L19.7	Intron	0.95	-0.06
46	cg04904300	Wiedman	EPIC	Chr22:20,076,641-20,076,641	DGCR8	Body	0.95	-
47	cg04917373	Nannini	EPIC	Chr1:231,489,251-231,489,301	SPRTN	3'UTR	0.95	-0.09
48	cg05009104	Nannini	EPIC	Chr7:45,002,980-45,002,980	MYO1G	Body	0.84	-

Table S2. Cannabis-associated CpG sites identified in previous studies (n=246). (The 9 cannabis-associated CpG sites that replicated across our tests of group comparisons and our fully covariate-adjusted tests of dose-response associations are shaded in gray.)

	CpG Site	Study	BeadChip	Genomic Location	Nearest Gene	Location Relative to Nearest Gene	Mean Age-45 Dunedin β	Test-Retest Reliability ICC (450k-EPIC)[a]
49	cg05086879	Nannini	EPIC	Chr22:39,861,490-39,861,490	MGAT3	5'UTR	0.88	-
50	cg05161803	Nannini	EPIC	Chr6:12,393,258-12,393,258	RNU6-48P	TSS1500	0.87	-
51	cg05169160	Nannini	EPIC	Chr2:178,484,438-178,484,488	TTC30A	Intergenic	0.96	0.05
52	cg05208619	Nannini	EPIC	Chr3:13,252,098-13,252,098	IQSEC1	5'UTR	0.93	-
53	cg05575921	Garrett<br>Nannini<br>Osborne	450k/EPIC<br>EPIC<br>EPIC	Chr5:373,377-373,427	AHRR	Intron	0.88	0.88
54	cg05615552	Nannini	EPIC	Chr6:33,160,903-33,160,953	RXRB	Intergenic	0.03	0.00
55	cg05689028	Nannini	EPIC	Chr11:118,086,759-118,086,809	AMICA1	Intron	0.94	0.04
56	cg05731136	Nannini	EPIC	Chr11:122,571,799-122,571,799	UBASH3B	Body	0.89	-
57	cg05922265	Nannini	EPIC	Chr5:120,097,605-120,097,655	CTD-2334D19.1	Intergenic	0.87	0.02
58	cg05998850	Nannini	EPIC	Chr2:216,981,058-216,981,108	XRCC5	Intron	0.92	0.06
59	cg06221963	Markunas	450K	Chr1:154,839,764-154,839,814	KCNN3	Intron	0.72	0.98
60	cg06300152	Nannini	EPIC	Chr1:150,268,771-150,268,771	MIRPS21	Body	0.95	-
61	cg06326914	Nannini	EPIC	Chr15:35,280,871-35,280,921	ZNF770	Intergenic	0.05	-0.04
62	cg06466031	Nannini	EPIC	Chr19:37,329,447-37,329,497	ZNF790	Intron	0.08	0.00
63	cg06581729	Nannini	EPIC	Chr2:29,004,756-29,004,756	PPP1CB	Body	0.94	-
64	cg06681167	Nannini	EPIC	Chr2:67,828,263-67,828,263	LINC02831	Body	0.95	-
65	cg06716419	Nannini	EPIC	Chr4:188,097,918-188,097,968	RP11-308K2.1	Intergenic	0.91	0.21
66	cg06815210	Nannini	EPIC	Chr3:8,844,052-8,844,052	OXTR	Intergenic	0.90	-
67	cg06969469	Nannini	EPIC	Chr2:121,554,768-121,554,818	GLI2	Intron	0.97	0.08
68	cg07011775	Nannini	EPIC	Chr10:72,964,759-72,964,759	UNC5B	Intergenic	0.75	-
69	cg07033820	Markunas	450K	Chr1:32,707,161-32,707,211	MTMR9LP	TSS	0.19	0.48
70	cg07064251	Nannini	EPIC	Chr2:43,948,676-43,948,676	PLEKHH2	Body	0.96	-
71	cg07178006	Markunas	450K	Chr11:20,184,717-20,184,767	DBX1	Intergenic	0.23	0.27

Table S2. Cannabis-associated CpG sites identified in previous studies (n=246). (The 9 cannabis-associated CpG sites that replicated across our tests of group comparisons and our fully covariate-adjusted tests of dose-response associations are shaded in gray.)

	CpG Site	Study	BeadChip	Genomic Location	Nearest Gene	Location Relative to Nearest Gene	Mean Age-45 Dunedin β	Test-Retest Reliability ICC (450k-EPIC)[a]
72	cg07344661	Nannini	EPIC	Chr4:185,187,124-185,187,124	ENPP6	Intergenic	0.91	-
73	cg07992500	Markunas	450K	Chr2:37,896,534-37,896,584	CDC42EP3	Intron	0.74	0.86
74	cg08145617	Markunas	450K	Chr3:32,858,492-32,858,542	TRIM71	Intergenic	0.06	0.08
75	cg08153621	Nannini	EPIC	Chr19:53,561,392-53,561,442	ERVV-2	Intergenic	0.19	0.43
76	cg08390696	Markunas	450K	Chr13:99,405,101-99,405,151	SLC15A1	Intergenic	0.42	0.53
77	cg08688629	Markunas	450K	Chr10:134,972,931-134,972,981	KNDC1	Intergenic	0.75	0.60
78	cg08760398	Garrett	450K/EPIC	ChrX:119442971	FAM70A	Body	0.38	-
79	cg08795904	Nannini	EPIC	Chr22:50,695,545-50,695,595	MAPK12	Coding region	0.88	0.12
80	cg08839808	Nannini	EPIC	Chr6:156,983,303-156,983,353	RP11-230C9.1	Exon	0.64	0.80
81	cg08923376	Wiedman	EPIC	Chr7:64,149,928-64,149,928	ZNF107	5'UTR	0.89	-
82	cg09040721	Nannini	EPIC	Chr6:41,658,881-41,658,881	TFEB	Body	0.95	-
83	cg09132256	Nannini	EPIC	Chr16:84,535,907-84,535,957	TLDC1	Intron	0.74	0.09
84	cg09254142	Nannini	EPIC	Chr1:25,943,842-25,943,892	MAN1C1	Intergenic	0.03	0.02
85	cg09393453	Nannini	EPIC	Chr19:54,224,373-54,224,423	MIR517B	Exon	0.64	-0.07
86	cg09501516	Nannini	EPIC	Chr1:163,039,154-163,039,204	RGS4	5'UTR	0.08	0.04
87	cg09607178	Nannini	EPIC	Chr6:29,978,431-29,978,481	ZNRD1-AS1	Intron	0.84	-0.02
88	cg09706133	Nannini	EPIC	Chr15:68,659,709-68,659,759	ITGA11	Intron	0.55	0.41
89	cg09825346	Nannini	EPIC	Chr1:161,718,565-161,718,615	DUSP12	Intergenic	0.90	0.07
90	cg09935388	Nannini	EPIC	Chr1:92,947,587-92,947,637	GFI1	Intron	0.83	0.75
91	cg09959525	Nannini	EPIC	Chr2:161,133,880-161,133,880	RBMS1	ExonBnd	0.80	-
92	cg10010780	Markunas	450K	Chr4:187,629,519-187,629,569	FAT1	Coding region	0.95	-0.01
93	cg10138977	Nannini	EPIC	Chr2:131,421,863-131,421,913	POTEJ	Intergenic	0.80	0.47
94	cg10260220	Nannini	EPIC	Chr17:40,811,109-40,811,159	TUBG2	Intergenic	0.06	-0.05
95	cg10270293	Nannini	EPIC	Chr16:4,654,665-4,654,715	C16orf96	Intergenic	0.74	0.16
96	cg10328583	Markunas	450K	Chr1:6,551,072-6,551,122	PLEKHG5	Intron	0.18	0.56

Table S2. Cannabis-associated CpG sites identified in previous studies (n=246). (The 9 cannabis-associated CpG sites that replicated across our tests of group comparisons and our fully covariate-adjusted tests of dose-response associations are shaded in gray.)								
	CpG Site	Study	BeadChip	Genomic Location	Nearest Gene	Location Relative to Nearest Gene	Mean Age-45 Dunedin β	Test-Retest Reliability ICC (450k-EPIC)[a]
97	cg10341310	Nannini	EPIC	Chr8:66,582,205-66,582,255	MTFR1	TSS	0.70	0.81
98	cg10586870	Markunas	450K	Chr5:75,722,316-75,722,366	IQGAP2	Intron	0.46	0.73
99	cg10616121	Nannini	EPIC	Chr11:111,895,340-111,895,390	DLAT	Intergenic	0.06	-0.02
100	cg10620881	Nannini	EPIC	Chr7:158,280,479-158,280,529	PTPRN2	Intron	0.92	0.40
101	cg10721491	Nannini	EPIC	Chr19:29,581,783-29,581,833	CTD-2081K17.2	Intergenic	0.95	0.07
102	cg10884287	Nannini	EPIC	Chr9:140,301,662-140,301,662	EXD3	Body	0.04	-
103	cg11035992	Nannini	EPIC	Chr17:7,657,424-7,657,474	DH2	Intron	0.96	0.09
104	cg11175241	Nannini	EPIC	Chr12:32,669,224-32,669,224	FGD4	5'UTR	0.69	-
105	cg11367172	Nannini	EPIC	Chr7:55,605,450-55,605,450	VOPP1	TSS	0.33	-
106	cg11518846	Nannini	EPIC	Chr6:133,562,245-133,562,295	EYA4	Intron	0.09	0.09
107	cg11588001	Nannini	EPIC	Chr19:29,704,239-29,704,289	UQCRFS1	3'UTR	0.11	0.08
108	cg12028375	Nannini	EPIC	Chr3:66,534,318-66,534,318	LRIG1	Body	0.92	-
109	cg12084925	Nannini	EPIC	Chr6:33,130,647-33,130,697	COL11A2	5'UTR	0.93	0.15
110	cg12211703	Nannini	EPIC	Chr3:42,204,097-42,204,097	TRAK1	Body	0.60	-
111	cg12438576	Nannini	EPIC	Chr11:89,232,167-89,232,217	RP11-745I13.1	Exon	0.85	0.38
112	cg12475321	Nannini	EPIC	Chr3:34,711,184-34,711,184	LINC01811	Intron	0.90	-
113	cg12510044	Nannini	EPIC	Chr22:22,115,473-22,115,473	MAPK1	3'UTR	0.82	-
114	cg12652442	Markunas	450K	Chr13:36,738,071-36,738,121	SOHLH2	Intergenic	0.88	0.45
115	cg12710152	Markunas	450K	Chr1:32,716,724-32,716,774	LCK	Intergenic	0.09	0.05
116	cg12992554	Nannini	EPIC	Chr1:17,046,814-17,046,864	ESPNP	Intergenic	0.87	0.81
117	cg13113737	Markunas	450K	Chr1:8,411,025-8,411,075	RERE	Intergenic	0.88	0.11
118	cg13179084	Nannini	EPIC	Chr12:116,638,602-116,638,602	MED13L	Body	0.93	-
119	cg13210467	Markunas	450K	Chr7:99,775,442-99,775,492	STAG3	TSS	0.48	0.46
120	cg13337507	Nannini	EPIC	Chr18:3,050,831-3,050,831	MYOM1	Intergenic	0.80	-
121	cg13444775	Nannini	EPIC	Chr11:124,546,591-124,546,591	SPA17	Body	0.25	-

Table S2. Cannabis-associated CpG sites identified in previous studies (n=246). (The 9 cannabis-associated CpG sites that replicated across our tests of group comparisons and our fully covariate-adjusted tests of dose-response associations are shaded in gray.)								
	CpG Site	Study	BeadChip	Genomic Location	Nearest Gene	Location Relative to Nearest Gene	Mean Age-45 Dunedin β	Test-Retest Reliability ICC (450k-EPIC)[a]
122	cg13552867	Nannini	EPIC	Chr1:160,053,688-160,053,738	KCNJ9	5'UTR	0.10	0.11
123	cg13810485	Nannini	EPIC	Chr5:58,440,035-58,440,035	PDE4D	Body	0.84	-
124	cg13966609	Nannini	EPIC	Chr11:117,352,681-117,352,731	DSCAML1	Coding region	0.99	-0.13
125	cg14219071	Nannini	EPIC	Chr2:48,996,041-48,996,041	STON1-GTF2A1L	Body	0.21	-
126	cg14223646	Nannini	EPIC	Chr7:155,855,650-155,855,700	AC021218.2	Intergenic	0.88	0.06
127	cg14237301	Fang	450K/EPIC	Chr16:28,506,428-28,506,478	APOBR	Coding region	0.77	0.60
128	cg14374923	Nannini	EPIC	Chr6:33,145,906-33,145,956	COL11A2	Coding region	0.86	0.37
129	cg14500389	Nannini	EPIC	Chr2:136,595,446-136,595,446	LCT	TSS	0.88	-
130	cg15088912	Nannini	EPIC	Chr13:48,987,465-48,987,465	LPAR6	1stExon	0.73	-
131	cg15204119	Nannini	EPIC	Chr19:10,613,179-10,613,229	KEAP1	3'UTR	0.08	0.06
132	cg15391425	Nannini	EPIC	Chr1:3,106,945-3,106,995	PRDM16	Intron	0.92	0.10
133	cg15412087	Nannini	EPIC	Chr11:120,085,144-120,085,194	OAF	Intron	0.92	0.05
134	cg15425280	Nannini	EPIC	Chr4:158,141,443-158,141,493	GRIA2	5'UTR	0.17	0.26
135	cg15540553	Nannini	EPIC	Chr11:64,018,642-64,018,692	PLCB3	Intergenic	0.09	0.02
136	cg15607642	Nannini	EPIC	Chr6:107,391,588-107,391,638	BEND3	Coding region	0.94	0.05
137	cg15627771	Nannini	EPIC	Chr2:192,111,084-192,111,084	MYO1B	5'UTR	0.57	-
138	cg15802887	Nannini	EPIC	Chr1:32,420,496-32,420,496	PTP4A2	Intergenic	0.43	-
139	cg16066871	Nannini	EPIC	Chr10:133,208,437-133,208,487	TCERG1L	Intergenic	0.94	0.11
140	cg16276224	Nannini	EPIC	Chr11:3,647,037-3,647,087	TRPC2	Intron	0.92	0.21
141	cg16504439	Nannini	EPIC	Chr4:20,647,245-20,647,245	SLIT2	Intergenic	0.83	-
142	cg16609878	Nannini	EPIC	Chr10:96,328,888-96,328,888	HELLS	5'UTR	0.94	-
143	cg16664193	Nannini	EPIC	Chr17:35,227,045-35,227,095	RP11-445F12.1	Intron	0.48	0.39
144	cg16674248	Nannini	EPIC	Chr22:46,664,401-46,664,451	TTC38	Coding region	0.93	0.21
145	cg16700555	Markunas	450K	Chr19:29,700,811-29,700,861	UQCRFS1	Intron	0.92	0.37
146	cg16736717	Nannini	EPIC	Chr7:54,397,759-54,397,759	LINC01445	TSS	0.94	-

Table S2. Cannabis-associated CpG sites identified in previous studies (n=246). (The 9 cannabis-associated CpG sites that replicated across our tests of group comparisons and our fully covariate-adjusted tests of dose-response associations are shaded in gray.)

	CpG Site	Study	BeadChip	Genomic Location	Nearest Gene	Location Relative to Nearest Gene	Mean Age-45 Dunedin β	Test-Retest Reliability ICC (450k-EPIC)[a]
147	cg16903225	Markunas	450K	Chr7:32,111,017-32,111,067	PDE1C	TSS	0.35	0.57
148	cg16947168	Nannini	EPIC	Chr3:62,651,287-62,651,287	CADPS	Body	0.91	-
149	cg16960213	Nannini	EPIC	Chr15:43,432,580-43,432,580	TMEM62	Body	0.91	-
150	cg17024593	Markunas	450K	Chr12:34,490,066-34,490,116	RP13-7D7.1	Intergenic	0.88	0.77
151	cg17218147	Nannini	EPIC	Chr3:38,995,416-38,995,416	SCN11A	TSS	0.79	-
152	cg17250160	Markunas	450K	Chr6:156,919,762-156,919,812	RP11-230C9.1	Intergenic	0.43	0.73
153	cg17285328	Wiedman	EPIC	Chr5:171,834,167-171,834,167	SH3PXD2B	Body	0.96	-
154	cg17301216	Markunas	450K	Chr15:89,920,299-89,920,349	LINC00925	Intron	0.43	0.70
155	cg17350345	Nannini	EPIC	Chr9:137,558,909-137,558,909	COL5A1	Body	0.14	-
156	cg17362109	Markunas	450K	Chr3:194,981,273-194,981,323	XXYLT1	Intron	0.09	-0.01
157	cg17456749	Nannini	EPIC	Chr12:111,807,010-111,807,060	RP3-473L9.4	Intergenic	0.02	0.04
158	cg17464820	Markunas	450K	Chr11:116,838,368-116,838,418	SIK3	Intron	0.74	0.67
159	cg17544636	Nannini	EPIC	Chr17:30,345,789-30,345,789	LRRC37B	Intron	0.90	-
160	cg17739917	Osborne	EPIC	Chr17:38,477,572-38,477,572	RARA	5'UTR	0.51	-
161	cg17902007	Nannini	EPIC	Chr13:101,184,798-101,184,848	GGACT	3'UTR	0.98	0.01
162	cg17968304	Nannini	EPIC	Chr20:11,916,902-11,916,902	BTBD3	Intergenic	0.90	-
163	cg18110140	Nannini	EPIC	Chr15:75,350,380-75,350,380	PPCDC	Intergenic	0.60	-
164	cg18211706	Nannini	EPIC	Chr12:54,867,283-54,867,333	GTSF1	TSS	0.76	0.48
165	cg18319941	Nannini	EPIC	Chr7:64,894,860-64,894,910	ZNF92	Intergenic	0.95	0.93
166	cg18387338	Nannini	EPIC	Chr7:26,591,438-26,591,438	LINC03095	Exon	0.86	-
167	cg18461093	Nannini	EPIC	Chr12:78,048,038-78,048,088	RP1-34H18.1	Intergenic	0.74	0.14
168	cg18489009	Nannini	EPIC	Chr19:46,056,722-46,056,772	OPA3	5'UTR	0.94	0.07
169	cg18880190	Nannini	EPIC	Chr15:40,399,609-40,399,609	BMF	5'UTR	0.82	-
170	cg18881764	Nannini	EPIC	Chr1:36,023,878-36,023,928	NCDN	Exon	0.06	0.00
171	cg19089201	Nannini	EPIC	Chr7:45,002,238-45,002,288	MYO1G	5'UTR	0.87	0.78

Table S2. Cannabis-associated CpG sites identified in previous studies (n=246). (The 9 cannabis-associated CpG sites that replicated across our tests of group comparisons and our fully covariate-adjusted tests of dose-response associations are shaded in gray.)								
	CpG Site	Study	BeadChip	Genomic Location	Nearest Gene	Location Relative to Nearest Gene	Mean Age-45 Dunedin β	Test-Retest Reliability ICC (450k-EPIC)[a]
172	cg19402405	Markunas	450K	Chr10:64,576,513-64,576,563	EGR2	Intron	0.04	0.03
173	cg19414984	Nannini	EPIC	Chr15:52,079,949-52,079,949	TMOD2	Body	0.86	-
174	cg19569686	Nannini	EPIC	Chr19:1,555,092-1,555,142	MEX3D	5'UTR	0.95	-0.07
175	cg19580944	Nannini	EPIC	Chr5:25,569,450-25,569,450	ENSG00000248605	Intergenic	0.84	-
176	cg19583287	Nannini	EPIC	Chr3:186,205,811-186,205,811	LINC02052	Body	0.93	-
177	cg19772897	Markunas	450K	Chr18:13,263,139-13,263,189	LDLRAD4	Intron	0.93	0.13
178	cg19856593	Nannini	EPIC	Chr21:38,064,187-38,064,237	AP000697.6	Intergenic	0.52	0.13
179	cg19857151	Nannini	EPIC	Chr1:235,985,453-235,985,503	LYST	Intron	0.94	0.04
180	cg20145687	Nannini	EPIC	Chr3:16,461,636-16,461,636	RFTN1	Body	0.89	-
181	cg20207973	Nannini	EPIC	Chr22:38,506,663-38,506,713	BAIAP2L2	TSS	0.93	0.08
182	cg20299935	Nannini	EPIC	Chr17:21,795,942-21,795,992	RP11-1109M24.5	Intron	0.84	0.91
183	cg20424400	Nannini	EPIC	Chr12:110,026,736-110,026,786	MVK	Exon	0.91	0.08
184	cg20777378	Wiedman	EPIC	Chr3:184,387,942-184,387,942	MAGEF1	Intergenic	0.92	-
185	cg20958467	Nannini	EPIC	Chr6:2,876,805-2,876,805	SERPINB9P1	TSS	0.63	-
186	cg20985028	Nannini	EPIC	Chr1:1,391,006-1,391,056	ATAD3C	Intron	0.94	0.11
187	cg21161138	Nannini	EPIC	Chr5:399,311-399,361	AHRR	Intron	0.82	0.49
188	cg21170085	Nannini	EPIC	Chr9:137,645,681-137,645,731	COL5A1	Coding region	0.95	0.42
189	cg21171274	Nannini	EPIC	Chr15:52,539,081-52,539,081	MYO5C	Body	0.44	-
190	cg21234547	Nannini	EPIC	Chr3:42,236,831-42,236,831	TRAK1	Body	0.93	-
191	cg21263605	Nannini	EPIC	Chr1:166,818,685-166,818,735	POGK	Coding region	0.93	0.69
192	cg21352345	Nannini	EPIC	Chr14:73,129,354-73,129,354	DPF3	3'UTR	0.94	-
193	cg21392402	Nannini	EPIC	Chr14:91,163,691-91,163,691	TTC7B	Body	0.94	-
194	cg21426547	Nannini	EPIC	Chr7:34,697,678-34,697,678	NPSR1-AS1	TSS	0.86	-
195	cg21447227	Nannini	EPIC	Chr7:158,656,628-158,656,678	WDR60	Intron	0.09	0.03
196	cg21491555	Nannini	EPIC	Chr22:19,967,785-19,967,835	ARVCF	Exon	0.92	0.03

Table S2. Cannabis-associated CpG sites identified in previous studies (n=246). (The 9 cannabis-associated CpG sites that replicated across our tests of group comparisons and our fully covariate-adjusted tests of dose-response associations are shaded in gray.)								
	CpG Site	Study	BeadChip	Genomic Location	Nearest Gene	Location Relative to Nearest Gene	Mean Age-45 Dunedin β	Test-Retest Reliability ICC (450k-EPIC)[a]
197	cg21495609	Nannini	EPIC	Chr11:69,080,688-69,080,688	MYEOV	Intergenic	0.52	-
198	cg21566642	Osborne	EPIC	Chr2:233,284,612-233,284,662	AC068134.5	Intergenic	0.64	0.80
199	cg21756190	Nannini	EPIC	Chr1:203,200,109-203,200,109	CHIT1	TSS	0.94	-
200	cg21804814	Markunas	450K	Chr7:152,599,951-152,600,001	ACTR3B	Intergenic	0.95	-0.06
201	cg21852554	Nannini	EPIC	Chr7:75,993,585-75,993,585	YWHAG	Intergenic	0.30	-
202	cg21960184	Nannini	EPIC	Chr11:113,804,385-113,804,435	HTR3B	Intron	0.94	-0.09
203	cg21998512	Markunas	450K	Chr7:92,077,030-92,077,080	GATAD1	5'UTR	0.03	0.07
204	cg22112841	Garrett	450K/EPIC	Chr6:31,740,889-31,740,939	VWA7	Coding region	0.86	0.19
205	cg22124000	Nannini	EPIC	Chr11:105,115,332-105,115,332	CARD18	5'UTR	0.60	-
206	cg22528521	Nannini	EPIC	Chr19:1,424,765-1,424,815	DAZAP1	Intron	0.90	0.20
207	cg22572071	Fang	450K/EPIC	Chr6:47,074,333-47,074,383	GPR110	Intergenic	0.83	0.44
208	cg22798873	Nannini	EPIC	Chr12:133,365,225-133,365,275	GOLGA3	Intron	0.91	0.27
209	cg22835724	Markunas	450K	Chr2:205,125,461-205,125,511	AC009498.1	Intergenic	0.93	0.05
210	cg22878990	Nannini	EPIC	Chr3:16,477,718-16,477,718	RTFN1	Body	0.94	-
211	cg23079012	Garrett	450K/EPIC	Chr2:8,343,661-8,343,711	LINC00299	Intron	0.97	0.19
212	cg23268344	Nannini	EPIC	Chr4:3,265,714-3,265,764	MSANTD1	3'UTR	0.87	0.19
213	cg23314514	Nannini	EPIC	Chr14:104,852,774-104,852,824	RP11-260M19.1	Intergenic	0.89	0.15
214	cg23619365	Markunas	450K	Chr13:112,712,008-112,712,058	SNORD44	Intergenic	0.11	0.26
215	cg23714123	Nannini	EPIC	Chr10:60,228,238-60,228,288	BICC1	Intergenic	0.94	-0.01
216	cg23767840	Wiedman	EPIC	Chr17:19,174,073-19,174,123	EPN2	Intron	0.90	0.98
217	cg23901606	Nannini	EPIC	Chr3:122,695,455-122,695,455	SEMA5B	5'UTR	0.93	-
218	cg23916896	Nannini	EPIC	Chr5:368,755-368,805	AHRR	Intron	0.32	0.45
219	cg23932689	Nannini	EPIC	Chr12:110,173,904-110,173,904	FAM222A	5'UTR	0.80	-
220	cg24007376	Nannini	EPIC	Chr13:51,275,033-51,275,033	DLEU1	Intron	0.94	-
221	cg24339197	Nannini	EPIC	Chr12:121,093,576-121,093,626	CABP1	5'UTR	0.95	0.02

Table S2. Cannabis-associated CpG sites identified in previous studies (n=246). (The 9 cannabis-associated CpG sites that replicated across our tests of group comparisons and our fully covariate-adjusted tests of dose-response associations are shaded in gray.)								
	CpG Site	Study	BeadChip	Genomic Location	Nearest Gene	Location Relative to Nearest Gene	Mean Age-45 Dunedin β	Test-Retest Reliability ICC (450k-EPIC)[a]
222	cg24405700	Garrett	450K/EPIC	Chr12:655829	B4GALNT3	Gene body	0.21	
223	cg24483247	Nannini	EPIC	Chr4:5,890,410-5,890,460	CRMP1	Intron	0.10	0.06
224	cg24976193	Nannini	EPIC	Chr4:100,685,228-100,685,228	DAPP1	Intergenic	0.86	-
225	cg25069772	Nannini	EPIC	Chr6:12,867,090-12,867,140	PHACTR1	Intron	0.94	-0.03
226	cg25189904	Nannini	EPIC	Chr1:68,299,492-68,299,542	GNG12-AS1	Intron	0.52	0.71
227	cg25228746	Markunas	450K	Chr2:127,865,330-127,865,380	BIN1	Intergenic	0.09	0.31
228	cg25343008	Nannini	EPIC	Chr1:202,470,230-202,470,230	PPP1R12B	Body	0.85	-
229	cg25343246	Markunas	450K	Chr12:34,756,242-34,756,292	RP13-7D7.1	Intergenic	0.44	0.74
230	cg25453681	Markunas	450K	Chr19:44,100,642-44,100,692	ZNF576	5'UTR	0.08	0.06
231	cg25609954	Markunas	450K	Chr4:3,472,245-3,472,295	DOK7	Intron	0.92	0.04
232	cg25668922	Markunas	450K	Chr5:34,656,125-34,656,175	RAI14	Intergenic	0.10	0.05
233	cg25875683	Markunas	450K	ChrX:153,029,721-153,029,771	PLXNB3	TSS	0.50	-
234	cg26003997	Nannini	EPIC	Chr9:96,395,832-96,395,832	PHF2	Body	0.95	-
235	cg26286198	Nannini	EPIC	Chr20:48,812,610-48,812,660	CEBPB	Intergenic	0.84	0.24
236	cg26582338	Nannini	EPIC	Chr3:155,523,692-155,523,742	C3orf33	Intron	0.09	0.07
237	cg26764244	Nannini	EPIC	Chr1:68,299,510-68,299,560	GNG12-AS1	Intron	0.23	0.61
238	cg26867465	Nannini	EPIC	Chr3:119,399,275-119,399,325	COX17	Intergenic	0.96	0.15
239	cg26870460	Markunas	450K	Chr11:6,947,710-6,947,760	ZNF215	TSS	0.11	0.51
240	cg26924392	Nannini	EPIC	Chr17:75,238,748-75,238,798	RP11-285E9.6	Intergenic	0.92	0.83
241	cg26987911	Markunas	450K	Chr2:113,522,572-113,522,622	CKAP2L	Intergenic	0.07	-0.01
242	cg27055782	Nannini	EPIC	Chr11:2,397,437-2,397,487	CD81	5'UTR	0.93	0.00
243	cg27068206	Nannini	EPIC	Chr11:70,559,004-70,559,054	SHANK2	Intron	0.93	0.26
244	cg27168438	Nannini	EPIC	Chr3:28,283,019-28,283,069	CMC1	Intergenic	0.05	0.03
245	cg27209861	Nannini	EPIC	Chr1:6,978,798-6,978,798	CAMTA1	Body	0.54	-
246	cg27564939	Markunas	450K	Chr17:2,207,194-2,207,244	SRR	TSS	0.02	0.35

Table S2. Cannabis-associated CpG sites identified in previous studies (n=246). (The 9 cannabis-associated CpG sites that replicated across our tests of group comparisons and our fully covariate-adjusted tests of dose-response associations are shaded in gray.)

	CpG Site	Study	BeadChip	Genomic Location	Nearest Gene	Location Relative to Nearest Gene	Mean Age-45 Dunedin β	Test-Retest Reliability ICC (450k-EPIC)[a]

Note. Test-retest reliabilities were available only for CpG sites on the 450k and EPIC BeadChips. Dashes indicate data not available. a. Obtained from https://osf.io/83ucs/.

Table S3. Description of measures.			
**Substance Use Exposures**	**Description**	**N**	**M (SD)**
Long-term cannabis users, long-term tobacco users, cannabis/tobacco non-users, cannabis quitters, tobacco quitters	Groups were defined based on past-year diagnostic interviews for cannabis and tobacco dependence, as well as self-reported frequency of substance use. Past-year cannabis and tobacco dependencies were assessed with the Diagnostic Interview Schedule (DIS)(38, 39) following criteria for the Diagnostic and Statistical Manual of Mental Disorders (77, 78) at each assessment age from age 18-45. Study members self-reported the number of days (0-365) they used cannabis and the number of cigarettes smoked per day at each assessment from age 18-45.	Long-term cannabis users: N=74.<br>Lifelong cannabis/tobacco non-users: N=189.<br>Long-term tobacco users: N=57.<br>Cannabis quitters: N=50.<br>Tobacco quitters: N=148.	-
Persistence of regular cannabis use	At each of the 6 assessment waves (ages 18, 21, 26, 32, 38, and 45 years), regular cannabis use was defined as 4+ use-days per week, based on past-year self-reported number of days of cannabis use.	Persistence of regular cannabis use (i.e., 4+ days per week) from age 18-45 comprised those who never used cannabis (n=244), (ii) used but never regularly (n=450), (iii) used regularly at one wave (n=44), (iv) used regularly at two waves (n=26), (v) used regularly at three waves (n=26), and (vi) used regularly at four or more waves (n=27).	-
Persistence of tobacco dependence	At each of the 6 assessment waves (ages 18, 21, 26, 32, 38, and 45 years), past-year tobacco dependence was assessed with the Diagnostic Interview Schedule (38, 39) following criteria for the Diagnostic and Statistical Manual of Mental Disorders (77, 78).	Persistence of tobacco dependence comprised study members who (i) never smoked tobacco (n=411), (ii) smoked tobacco daily at one or more assessment waves but were never diagnosed with tobacco dependence (n=114), (iii) were diagnosed at one wave (n=92), (iv) were diagnosed at two waves (n=81), (v) were diagnosed at three waves (n=52), and (vi) were diagnosed at four or more waves (n=67).	-
**Outcome**			

Age-45 DNA Methylation	DNA methylation measurement is described in the main text and elsewhere (32).	818	Mean beta values for each CpG site are shown in Table S2.
**Covariates**			
Principal Components	Principal components analysis was performed on all available normalization control probes on the EPIC array using the R package '*pcaMethods.'* 32 principal components were required to explain 90% of the variation in control probe methylation values.	818	-
White Blood Cell Counts	Counts (10^9/L) of lymphocytes, monocytes, basophils, eosinophils, and neutrophils were made from whole blood drawn at the cohort's age-45 assessment during 2017-2019.	862	Lymphocytes: 2.32 (0.65)<br>Monocytes: 0.68 (0.20)<br>Basophils: 0.04 (0.03)<br>Eosinophils: 0.22 (0.23)<br>Neutrophils: 4.48 (1.41)
Childhood SES	The socioeconomic status of Study members' parents was measured with the Elley-Irving scale (79), the forerunner of the NZSEI-06, which assigned occupations into 1 of 6 SES groups (from 1 = unskilled laborer to 6 = professional). The higher of either parents' occupation was averaged spanning the period from Study members' birth to age 15 (1972-1987).	818	3.81 (1.13)
Childhood Low Self-Control	Assessed using a multi-occasion/multi-informant strategy, across ages 3-11 years. Nine measures of childhood self-control in the composite include observational ratings of children's lack of control, parent and teacher reports of impulsive aggression, and parent, teacher, and self-reports of hyperactivity, lack of persistence, inattention, and impulsivity (43).	818	-0.03 (0.94)
Family History of Substance Dependence	Family histories were collected from study members (when they were age 30-33 years) and from their parents. Family psychiatric history data were collected about each study member's biological parents, grandparents, and siblings. Each participant's family history of substance use disorder was calculated as the % of family members with a positive history of disorder, taking into account genetic relatedness. This variable is a quantitative dimension (80).	818	0.14 (0.16)
Persistence of Alcohol Dependence	At each of the 6 assessment waves (ages 18, 21, 26, 32, 38, and 45 years), past-year tobacco dependence was assessed with the Diagnostic Interview Schedule (38, 39) following criteria for the Diagnostic and Statistical Manual of Mental Disorders (77, 78).	Persistence of alcohol dependence comprised study members who (i) never used alcohol (n=47), (ii) drank alcohol at least weekly at one or more assessment waves but were never diagnosed with alcohol dependence (n=484), (iii)	-

		were diagnosed at one wave (n=152), (iv) were diagnosed at two waves (n= 68), (v) were diagnosed at three waves (n=40), and (vi) were diagnosed at four or more waves (n=24).	
Persistent Illicit Drug Dependence	Past-year dependence on illicit drugs other than cannabis was assessed from ages 26-45 years with the Diagnostic Interview Schedule (38, 39) following criteria for the Diagnostic and Statistical Manual of Mental Disorders (77, 78).	Persistent illicit drug dependence comprised study members who (i) diagnosed at two or more study waves with illicit drug dependence (n=20), and (ii) those who did not (n=798).	-

Table S4. Background characteristics and substance use for the full cohort and for long-term cannabis users, long-term tobacco users, former users, and non-users.

	Full Cohort (N=818)	Long-term Cannabis Users (N=74)	Lifelong Cannabis/ Tobacco Non-Users (N=189)	Long-term Tobacco Users (N=57)	Cannabis Quitters (N=50)	Tobacco Quitters (N=148)
**Background Characteristics**						
Sex, % Male (N)	50.61 (414)	67.57 (50)	39.68 (75)	40.35 (23)	60.00 (30)	44.59 (66)
Childhood SES, M (SD)	3.81 (1.13)	3.45 (1.13)	3.91 (1.15)	3.23 (0.98)	3.66 (1.18)	3.77 (1.08)
Childhood Low Self-Control, M (SD)	-0.03 (0.94)	0.34 (1.09)	-0.18 (0.87)	0.45 (1.09)	0.18 (1.13)	-0.09 (0.82)
Family History of Substance Dependence, M (SD)	0.14 (0.16)	0.21 (0.21)	0.09 (0.13)	0.18 (0.19)	0.19 (0.17)	0.17 (0.16)
**Age-45 Substance Use and Dependence**						
Cannabis (# of days of use in past-year), M (SD)	24.67 (0.81)	253.34 (119.19)	0.00 (0.00)	0.09 (0.47)[b]	0.00 (0.00)	23.65
Regular Cannabis Use,[a] % (N)	5.77 (47)	62.16 (46)	0.00 (0)	0.00 (0)	0.00 (0)	5.44 (8)
Cannabis Dependence, % (N)	1.84 (15)	20.27 (15)	0.00 (0)	0.00 (0)	0.00 (0)	2.04 (3)
Daily Tobacco Use, % (N)	19.49 (159)	66.22 (49)	0.00 (0)	100.00 (57)	26.00 (13)	0.00 (0)
Tobacco Dependence, % (N)	10.78 (88)	45.95 (34)	0.00 (0)	50.88 (29)	12.00 (6)	0.68 (1)
Weekly Alcohol Use, % (N)	93.01 (758)	91.89 (68)	90.48 (171)	89.47 (51)	84.00 (42)	93.88 (138)
Alcohol Dependence, % (N)	10.80 (88)	20.27 (15)	0.00 (0)	10.53 (6)	14.00 (7)	16.33 (24)
Illicit Drug Dependence, % (N)	2.09 (17)	13.51 (10)	0.00 (0)	0.00 (0)	2.00 (1)	0.68 (1)
**Lifetime Substance Use/Dependence**						
Regular Cannabis Use,[a] % (N)	15.06 (123)	89.19 (66)	0.00 (0)	0.00 (0)	54.00 (27)	20.27 (30)
Cannabis Dependence, % (N)	17.38 (142)	74.32 (55)	0.00 (0)	0.00 (0)	86.00 (43)	29.05 (43)
Tobacco Dependence, % (N)	34.74 (292)	83.78 (62)	0.00 (0)	85.96 (49)	60.00 (30)	100.00 (148)
Alcohol Dependence, % (N)	34.76 (284)	59.46 (44)	0.00 (0)	35.09 (20)	72.00 (36)	50.68 (75)
Illicit Drug Dependence, % (N)	5.51 (45)	31.08 (23)	0.00 (0)	0.00 (0)	10.00 (5)	4.05 (6)

Note. a. Regular cannabis use=4+ days per week. b. Two study members in the long-term tobacco use group had used cannabis use at age 45 for a maximum of 3 days in the past year.

Table S5. A comparison of cannabis-related CpG sites from tests of group comparisons and tests of dose-response associations.

Group Comparison: Long-term Cannabis Users vs. Cannabis/Tobacco Non-Users			Dose-Response Association: Exposure=Persistence of Regular Cannabis Use from Age 18-45					
Adjusted for Sex, Methylation Control Probe, and White Blood Cell Counts			Adjusted for Sex, Methylation Control Probe, and White Blood Cell Counts				Additionally Adjusted for Childhood Covariates and Persistent Use of Other Substances	
	Significant at α <.05	Significant at α adjusted for multiple testing	Significant at α <.05		Significant at α adjusted for multiple testing		Significant at α adjusted for multiple testing	
1	cg05575921	cg05575921	1	cg05575921	1	cg05575921	1	cg05575921
2	cg21566642	cg21566642	2	cg21566642	2	cg21566642	2	cg21566642
3	cg01940273	cg01940273	3	cg01940273	3	cg01940273	3	cg01940273
4	cg17739917	cg17739917	4	cg17739917	4	cg17739917	4	cg17739917
5	cg21161138	cg21161138	5	cg21161138	5	cg21161138	5	cg21161138
6	cg03636183	cg03636183	6	cg03636183	6	cg03636183	6	cg03636183
7	cg25189904	cg25189904	7	cg25189904	7	cg25189904		
8	cg18110140	cg18110140	8	cg18110140	8	cg18110140		
9	cg05086879	cg05086879	9	cg05086879	9	cg05086879	7	cg05086879
10	cg23079012	cg23079012	10	cg23079012	10	cg23079012	8	cg23079012
11	cg02978227	cg02978227	11	cg02978227	11	cg02978227	9	cg02978227
12	cg18387338	cg18387338	12	cg18387338	12	cg18387338		
13	cg09935388	cg09935388	13	cg09935388	13	cg09935388		
14	cg23916896	cg23916896	14	cg23916896	14	cg23916896		
15	cg05009104	cg05009104	15	cg05009104	15	cg05009104		
16	cg26286198	cg26286198	16	cg26286198				
17	cg15088912	cg15088912	17	cg15088912	16	cg15088912		

18	cg19089201		18	cg19089201				
19	cg17350345		19	cg17350345				
20	cg26764244		20	cg26764244				
21	cg22124000		21	cg22124000				
22	cg14219071		22	cg14219071				
23	cg12510044		23	cg12510044				
24	cg21852554							
25	cg16276224		24	cg16276224				
26	cg21491555							
27	cg15802887							
28	cg13966609							
29	cg18880190							
30	cg22572071		25	cg22572071				
31	cg04864586		26	cg04864586				
32	cg05922265							
33	cg03093806		27	cg03093806				
34	cg27055782							
35	cg08153621		28	cg08153621				
			29	cg10620881				
			30	cg23932689				
			31	cg05161803				
			32	cg17250160				
			33	cg23268344				
			34	cg00761236				
			35	cg05208619	17	cg05208619		
			36	cg02616769				
			37	cg17218147				
			38	cg01212491				
			39	cg13810485				
			40	cg14374923				
			41	cg06815210				
			42	cg03802952				

			43	cg23314514				
			44	cg03905975				
			45	cg13179084				
			46	cg01039752				
			47	cg01668099				
			48	cg24976193				
			49	cg07011775				
			50	cg00785657				
			51	cg09706133				
			52	cg19856593				

Table S6. Replication at age 38: a comparison of long-term cannabis users and cannabis/tobacco non-users at age 38 on age-38 DNA methylation. Results are shown for the 9 cannabis-related CpG sites at age 45 that replicated across tests of group comparisons and covariate-adjusted tests of dose-response associations.

		Group Means (SD)				Long-Term Cannabis Users vs. Cannabis/Tobacco Non-Users			
		Lifelong Cannabis/ Tobacco Non-Users (N=177)		Long-Term Cannabis Users (N=76)					
	Age-38 CpG	M	SD	M	SD	Adjusted Mean Difference	95% CI		p
1	cg05575921	0.62	0.25	-1.16	1.04	-2.44	-2.55	-2.33	**<.0001**±
2	cg21566642	0.64	0.54	-1.00	0.82	-1.51	-1.73	-1.28	**<.0001**±
3	cg03636183	0.47	0.53	-0.84	1.15	-1.03	-1.22	-0.84	**<.0001**±
4	cg21161138	0.47	0.62	-0.95	1.22	-1.18	-1.40	-0.95	**<.0001**±
5	cg01940273	0.58	0.67	-0.85	0.91	-1.30	-1.56	-1.05	**<.0001**±
6	cg17739917	-	-	-	-	-	-	-	-
7	cg05086879	-	-	-	-	-	-	-	-
8	cg02978227	-	-	-	-	-	-	-	-
9	cg23079012	0.27	0.55	-0.65	1.74	-0.32	-0.44	-0.19	**<.0001**±

Note. Means are crude means, unadjusted for covariates, and standardized (M=0.00, SD=1.00) on the representative cohort. Adjusted mean differences are mean differences from robust regression, adjusted for sex, methylation-array control probe principal components indexing technical variation, and white blood cell counts. Bold=statistically significant at α=.05. ±=statistically significant at α adjusted for six tests. Dashes=CpG site not present on age-38 450K BeadChip.

Table S7. A comparison of cannabis quitters with cannabis/tobacco non-users and long-term cannabis users. (Results are shown for the 9 CpG sites that survived adjustment for multiple testing in group comparisons and in fully covariate-adjusted tests of dose-response associations.)

	Group Means			Statistical Tests: Cannabis Quitters vs. Cannabis/Tobacco Non-Users				Statistical Tests: Cannabis Quitters vs. Long-term Cannabis Users			
CpG	Cannabis /Tobacco Non-Users (N=182)	Long-term Cannabis Users (N=73)	Cannabis Quitters (N=49)	Adjusted Mean Difference	95% CI		p	Adjusted Mean Difference	95% CI		p
1 cg05575921	0.57	-1.30	-0.33	-0.31	-0.46	-0.16	**<.0001**±	1.64	1.47	1.81	**<.0001**±
2 cg21566642	0.60	-1.12	-0.44	-0.86	-1.11	-0.61	**<.0001**±	0.86	0.57	1.14	**<.0001**±
3 cg03636183	0.42	-1.17	-0.38	-0.51	-0.70	-0.31	**<.0001**±	0.51	0.29	0.74	**<.0001**±
4 cg21161138	0.46	-1.02	-0.37	-0.57	-0.77	-0.36	**<.0001**±	0.57	0.33	0.81	**<.0001**±
5 cg01940273	0.54	-1.11	-0.49	-0.76	-1.01	-0.51	**<.0001**±	0.90	0.61	1.19	**<.0001**±
6 cg17739917	0.60	-1.13	-0.32	-0.60	-0.84	-0.37	**<.0001**±	1.01	0.74	1.28	**<.0001**±
7 cg05086879	0.34	-1.00	-0.25	-0.56	-0.80	-0.32	**<.0001**±	0.22	-0.06	0.49	0.1255
8 cg02978227	0.27	-0.62	-0.48	-0.33	-0.54	-0.11	**0.0033**±	0.40	0.15	0.65	**0.0017**±
9 cg23079012	0.28	-0.77	0.00	-0.08	-0.17	0.02	0.1168	0.24	0.13	0.35	**<.0001**±

Note. Group means are crude means, unadjusted for covariates. Means were standardized (M=0.00, SD=1.00) on the representative cohort. Statistical tests are from robust regression and are adjusted for sex, principal components indexing technical variation, and white blood cell counts. Bold=p<.05. ±Statistically significant after adjustment for multiple testing.

Table S8. A comparison of tobacco quitters with cannabis/tobacco non-users and long-term tobacco users. (Results are shown for the 17 CpG sites that survived adjustment for multiple testing in group comparisons and in fully covariate-adjusted tests of dose-response associations.)

					Statistical Tests							
	Group Means				Tobacco Quitters vs. Cannabis/Tobacco Non-Users				Tobacco Quitters vs. Long-Term Tobacco Users			
	CpG	Cannabis/Tobacco Non-Users (N=182)	Long-term Tobacco Users (N=56)	Tobacco Quitters (N=147)	Adjusted Mean Difference	95 % CI		p	Adjusted Mean Difference	95 % CI		p
1	cg05575921	0.57	-1.64	0.05	2.31	2.21	2.42	**<.0001**±	-0.34	-0.42	-0.27	**<.0001**±
2	cg21566642	0.60	-1.36	-0.12	1.44	1.24	1.65	**<.0001**±	-0.65	-0.79	-0.51	**<.0001**±
3	cg03636183	0.42	-1.35	0.00	1.15	0.98	1.32	**<.0001**±	-0.33	-0.45	-0.22	**<.0001**±
4	cg21161138	0.46	-1.38	0.09	1.38	1.19	1.57	**<.0001**±	-0.40	-0.54	-0.27	**<.0001**±
5	cg01940273	0.54	-1.38	-0.06	1.35	1.12	1.58	**<.0001**±	-0.67	-0.83	-0.52	**<.0001**±
6	cg17739917	0.60	-1.16	-0.03	1.48	1.28	1.69	**<.0001**±	-0.51	-0.66	-0.37	**<.0001**±
7	cg05086879	0.34	-1.14	0.14	1.00	0.78	1.23	**<.0001**±	-0.26	-0.42	-0.11	**0.0009**±
8	cg02978227	0.27	-0.67	-0.04	0.62	0.41	0.82	**<.0001**±	-0.24	-0.38	-0.10	**0.0008**±
9	cg23079012	0.28	-0.86	0.07	0.45	0.35	0.54	**<.0001**±	-0.09	-0.15	-0.02	**0.0065**±
10	cg18110140	0.38	-0.89	-0.09	0.71	0.44	0.98	**<.0001**±	-0.59	-0.77	-0.40	**<.0001**±
11	cg09935388	0.27	-0.96	0.07	0.80	0.60	1.00	**<.0001**±	-0.23	-0.37	-0.09	**0.0010**±
12	cg25189904	0.37	-0.95	-0.04	0.89	0.59	1.20	**<.0001**±	-0.41	-0.62	-0.21	**<.0001**±
13	cg05009104	-0.22	0.63	0.08	-0.60	-0.93	-0.28	**0.0003**±	0.22	0.00	0.45	**0.0495**
14	cg23916896	0.26	-0.58	-0.05	0.52	0.20	0.84	**0.0014**±	-0.32	-0.53	-0.10	**0.0046**
15	cg18387338	0.19	-0.78	0.01	0.78	0.51	1.05	**<.0001**±	-0.20	-0.38	-0.01	**0.0347**
16	cg15088912	0.12	-0.68	-0.04	0.45	0.18	0.72	**0.0011**±	-0.17	-0.36	0.01	0.0668
17	cg19089201	-0.20	0.63	0.04	-0.52	-0.82	-0.21	**0.0011**±	0.13	-0.08	0.35	0.2177

Note. Group means are crude means, unadjusted for covariates. Means were standardized (M=0.00, SD=1.00) on the representative cohort. Statistical tests are from robust regression and are adjusted for sex, principal components indexing technical variation, and white blood cell counts. Bold=p<.05.
±Statistically significant after adjustment for multiple testing.

Table S9. Dose-response associations between cannabis quit length and age-45 DNA methylation. Results are shown for the 9 cannabis-related CpG sites that replicated across tests of group comparisons and covariate-adjusted tests of dose-response associations.

		Adjusted for Covariates[a]			
	Age-45 CpG	Est	95% CI		p
1	cg05575921	0.37	0.21	0.53	**<.0001**±
2	cg21566642	0.28	0.15	0.40	**<.0001**±
3	cg03636183	0.29	0.10	0.10	**.0023**±
4	cg21161138	0.33	0.17	0.49	**<.0001**±
5	cg01940273	0.38	0.23	0.52	**<.0001**±
6	cg17739917	0.27	0.12	0.42	**.0005**±
7	cg05086879	0.30	0.11	0.49	**.0021**±
8	cg02978227	0.18	0.06	0.30	**.0033**±
9	cg23079012	0.11	-0.01	0.23	.0693

Note. The exposure is quit length -- a quantitative variable with values that ranged from 0-4 using data from long-term cannabis users (n=74) and cannabis quitters (n=49): (0) had not quit (i.e., age-45 long-term cannabis users; n=74), (1) quit by age 45 (n=17), (2) quit by age 38 (n=12), (3) quit by age 32 (n=10), (4) quit by age 21/26 (n=10). a. Covariates=sex, methylation-array control probe principal components indexing technical variation, white blood cell counts, childhood SES, low childhood self-control, family history of substance dependence, persistence of tobacco use, persistence of alcohol use, and persistent illicit drug dependence. Bold=p<.05. ±Statistically significant after adjustment for 9 tests.

Table S10. Summary of CpG sites that were consistently associated with cannabis use and tobacco use in group comparisons and in covariate-adjusted tests of dose-response associations, after adjusting for multiple testing (cannabis: n=9 CpG sites; tobacco: n=17 CpG sites).

CpG	Associated with Cannabis, Tobacco, or Both in Dunedin Study	Genomic Location	Nearest Coding Region	Nearest Gene Expression Patterns[a]	Location Relative to Nearest Gene	Selected Association Evidence[b]
1 cg05575921	Both	Chr5:373,377-373,427	AHRR	Widespread. Very high in testes. Low in WBC and lung.	Intron	Smoking (81); lung cancer (9); alcohol consumption (45); FEV1/FVC/lung function (82); serum cotinine (83); maternal smoking (84); PTSD (85)
2 cg21566642	Both	Chr2:233,284,612-233,284,662	ALPG	Unprocessed pseudogene.	Intergenic	Smoking (81); lung cancer (9); alcohol consumption (86); FEV1/FVC (87); serum cotinine (83)
3 cg03636183	Both	Chr19:17,000,536-17,000,586	F2RL3	Widespread. High in lung. Low in leukocytes.	Coding region	Smoking (81); lung cancer (9); alcohol consumption (86); FEV1/FVC (87); serum cotinine (83)
4 cg21161138	Both	Chr5:399,311-399,361	AHRR	Widespread. Very high in testes. Low in WBC and lung.	Intron	Smoking (81); lung cancer (9); FEV1/FVC (87); serum cotinine (83); maternal smoking (84)
5 cg01940273	Both	Chr2:233,284,885-233,284,935	ALPG	Lung. Fallopian tubes. Placenta.	Intergenic	Smoking (81); lung cancer (9); alcohol consumption (45); FEV1/FVC (87); serum cotinine (83); gestational age (88); PTSD (89); e-cigarette use (90); cognitive abilities (91)
6 cg17739917	Both	Chr17:038,477,572	RARA	Widespread. Basic cellular process-related transcript.	Intron	Smoking (70); lung cancer (46)
7 cg05086879	Both	Chr22:039,861,490	MGAT3	Brain, intestine	Intron	Smoking (70); lung cancer (46)
8 cg02978227	Both	Chr3:098,292,027	CPOX	Widespread. Highest in erythroid cells (oxygen transport).	Intergenic	Smoking (70); atopy (92)
9 cg23079012	Both	Chr2:8,343,661-8,343,711	LINC00299	-	Intron	Smoking (70)
10 cg18110140	Tobacco	Chr15:075,350,380	PPCDC	Widespread. Non-specific expression.	Intergenic	Smoking (70); e-cigarette use (90)
11 cg09935388	Tobacco	Chr1:92,947,587-92,947,637	GFI1	High expression in bone marrow and lymphoid tissues/immune cell response.	Intron	Smoking (81); lung cancer (46); alcohol consumption (86); FEV1/FVC (87); serum cotinine (83); maternal smoking (84); cognitive abilities (91)

Table S10. Summary of CpG sites that were consistently associated with cannabis use and tobacco use in group comparisons and in covariate-adjusted tests of dose-response associations, after adjusting for multiple testing (cannabis: n=9 CpG sites; tobacco: n=17 CpG sites).

CpG	Associated with Cannabis, Tobacco, or Both in Dunedin Study	Genomic Location	Nearest Coding Region	Nearest Gene Expression Patterns[a]	Location Relative to Nearest Gene	Selected Association Evidence[b]
12 cg25189904	Tobacco	Chr1:68,299,492-68,299,542	GNG12-AS1	Widespread. Highest in digestive tract. Non-specific expression.	Intron	Smoking (81); alcohol consumption (86); serum cotinine (83); maternal smoking (84); gestational age (93); PTSD (89)
13 cg05009104	Tobacco	Chr7:045,002,980	MYO1G	Widespread. Highest in immune cells, bone marrow and lymphoid tissue, then lung.	Body	Smoking (70)
14 cg23916896	Tobacco	Chr5:368,755-368,805	AHRR	Widespread. Very high in testes. Low in WBC and lung.	Intron	Smoking (81); serum cotinine (83); maternal smoking (84)
15 cg18387338	Tobacco	Chr7:026,591,438	LINC03095	Non-protein coding RNA.	Intergenic	Smoking (46)
16 cg15088912	Tobacco	Chr13:048,987,465	LPAR6; RB1	Both genes widely expressed with low tissue specificity.	1stExon	Smoking (70); lung cancer (94)
17 cg19089201	Tobacco	Chr7:45,002,238-45,002,288	MYO1G	Lung, bone marrow, lymphoid tissues	Intron	Smoking (81); maternal smoking (84)

Note. CpG sites were selected if they survived adjustment for multiple testing in group comparisons and in covariate-adjusted tests of dose-response associations. a. Taken from Human Protein Atlas. b. Taken from MRC-IEU EWAS Catalog (95). WBC=white blood cells.

Figure S1. Attrition analyses.

We conducted an attrition analysis using childhood SES, childhood IQ, and history of psychopathology to determine whether participants in the Phase 45 data collection were representative of the original cohort. We report childhood SES and childhood IQ because they are known to be strong predictors of late-life health outcomes, as shown by many cohort studies from many nations. Childhood SES and childhood IQ separately predict health and social outcomes in adulthood, and these outcomes include physical functions, cognitive decline, mental health, inflammation, metabolic syndrome, disease incidence, dementia, mortality, and also neuroimaging-based, genomic, and epigenetic indicators of health. Based on the literature, we report three groups: Study members who died before age 45 and thus could not have taken part in data collection, Study members who were alive and thus could take part, and Study members who actually did take part. We compared these three groups to the original birth cohort. The figures below show that the small group of study members who had died before age 45 had significantly lower mean childhood IQ on average as a group, and somewhat lower mean childhood SES. (Bars represent the range of values in the cohort.) Some of the early deaths were Dunedin Study members who had more disadvantages in their lives leading to poorer health and increased risk of early mortality. Study members who died of childhood diseases may have been already unwell at the time of IQ testing, which could have lowered their scores. However, cohort members who are still alive and cohort members who took part in data collection did not differ from the full original cohort on their mean childhood IQ and SES; they still represent population variation on these key health risk factors.

No significant differences in childhood SES were found between the full cohort, those deceased, those still alive, or those seen at Phase 45.

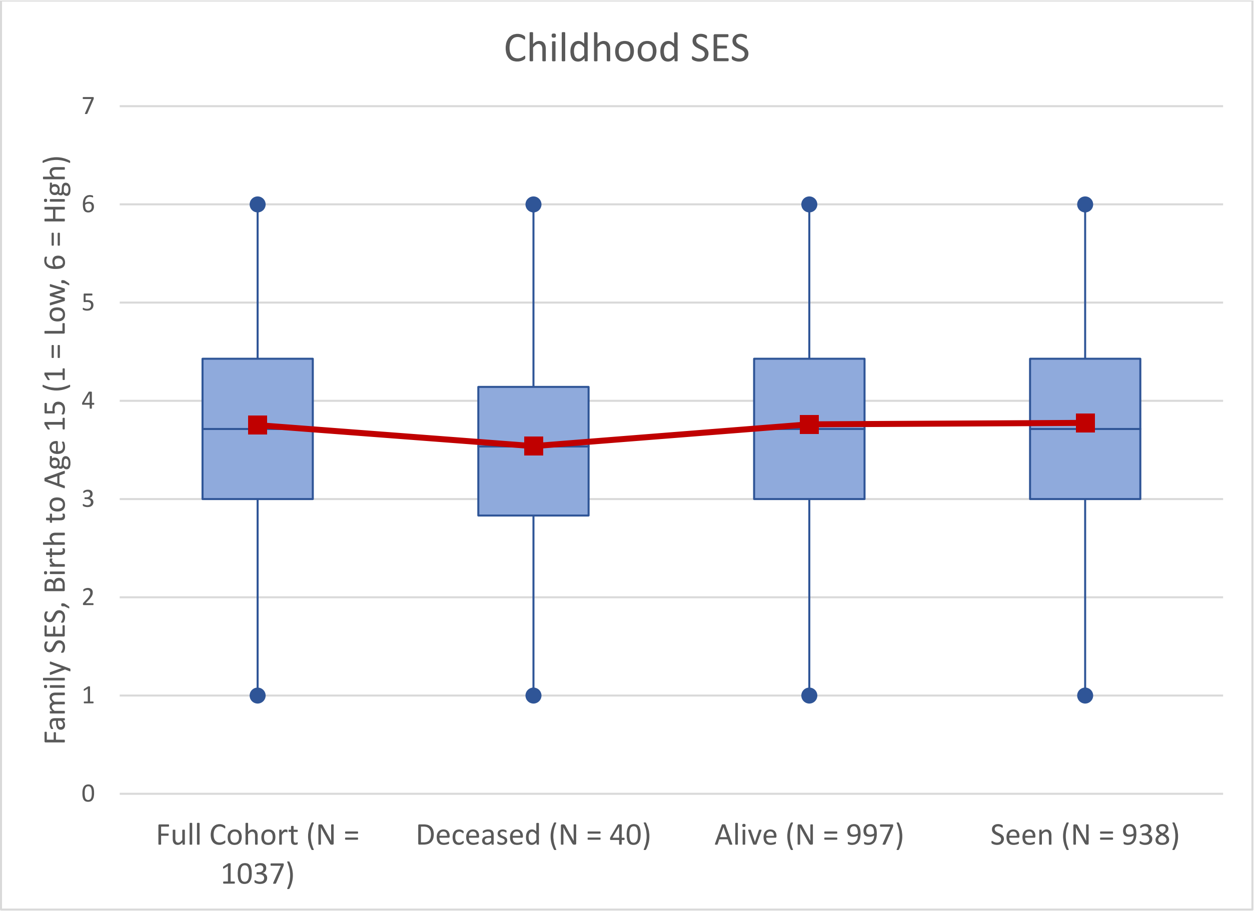

No significant differences in childhood IQ were found between the full cohort, those still alive, or those seen at Phase 45. Those who were deceased by the Phase 45 data collection had significantly lower childhood IQ than those who were still alive (t = 2.09, p = .04).

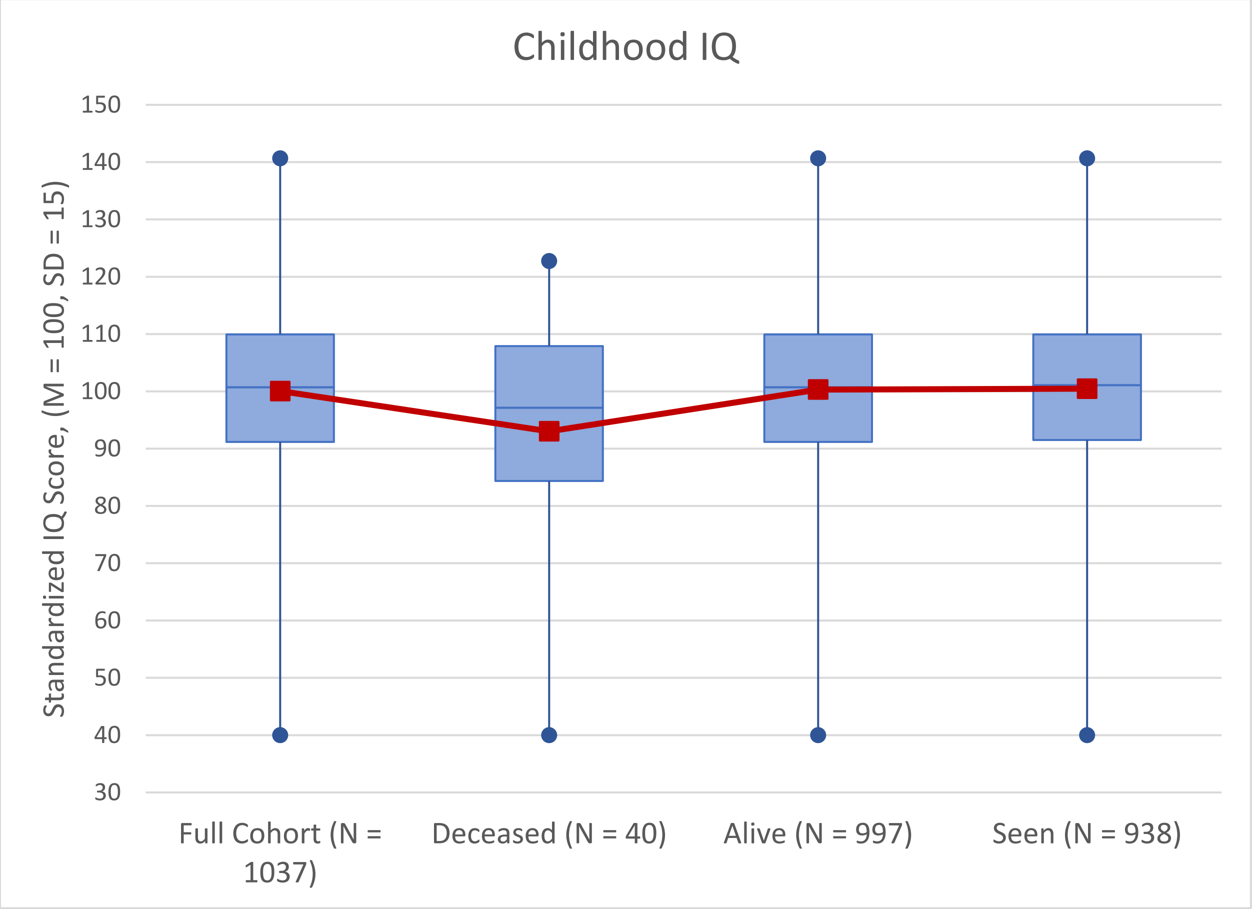

No significant differences in history of psychopathology were found between the full cohort, those still alive, or those seen at Phase 45. Those who were deceased by the Phase 45 data collection had significantly more extensive histories of psychopathology (i.e.,higher p-factor scores) than those who were still alive (t = -2.86, p = .004).

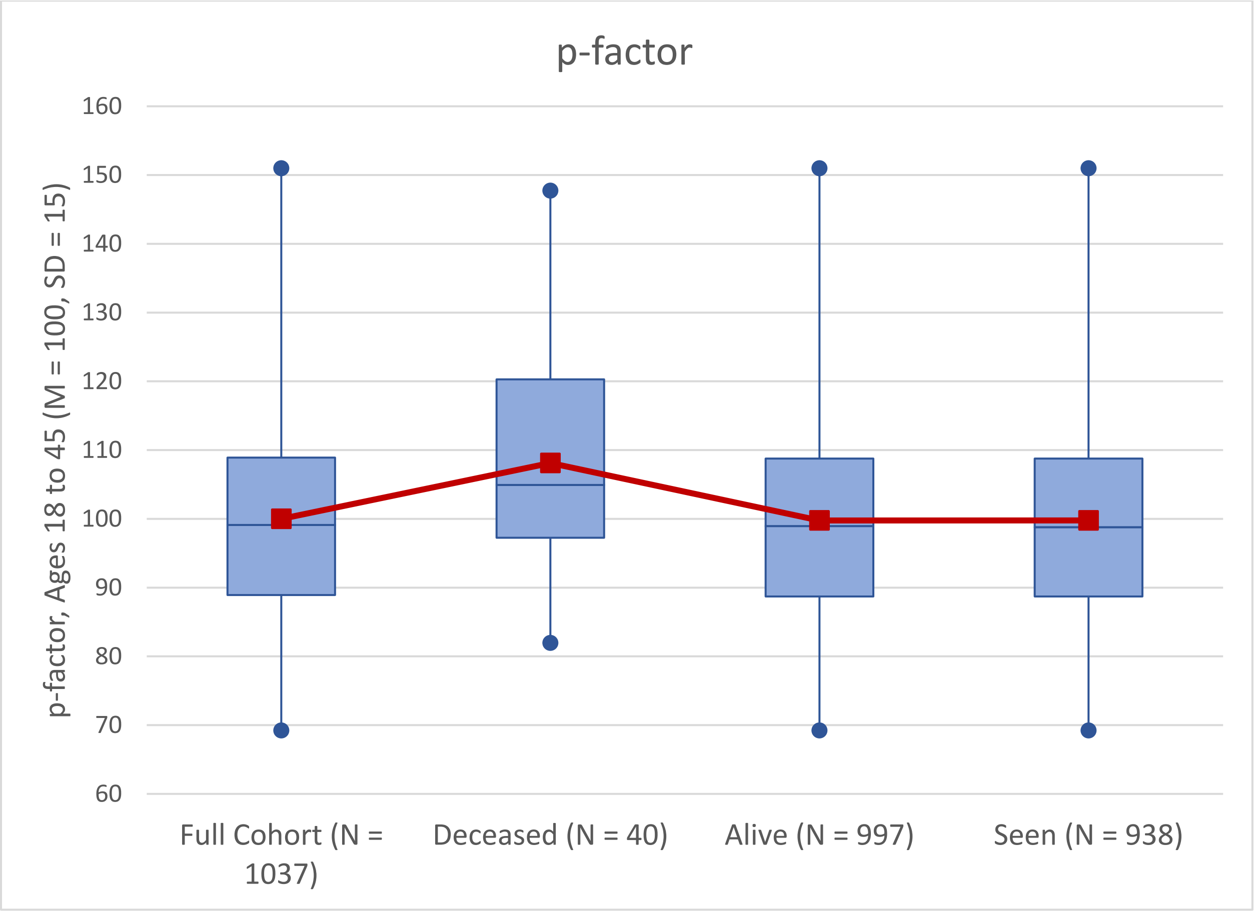

Figure S2. Groups for qualitative comparisons: Long-term cannabis users (N=74), Long-term Tobacco Users (N=57), Lifelong Cannabis/Tobacco Non-users (N=189), Cannabis Quitters (N=50), Tobacco Quitters (N=148).

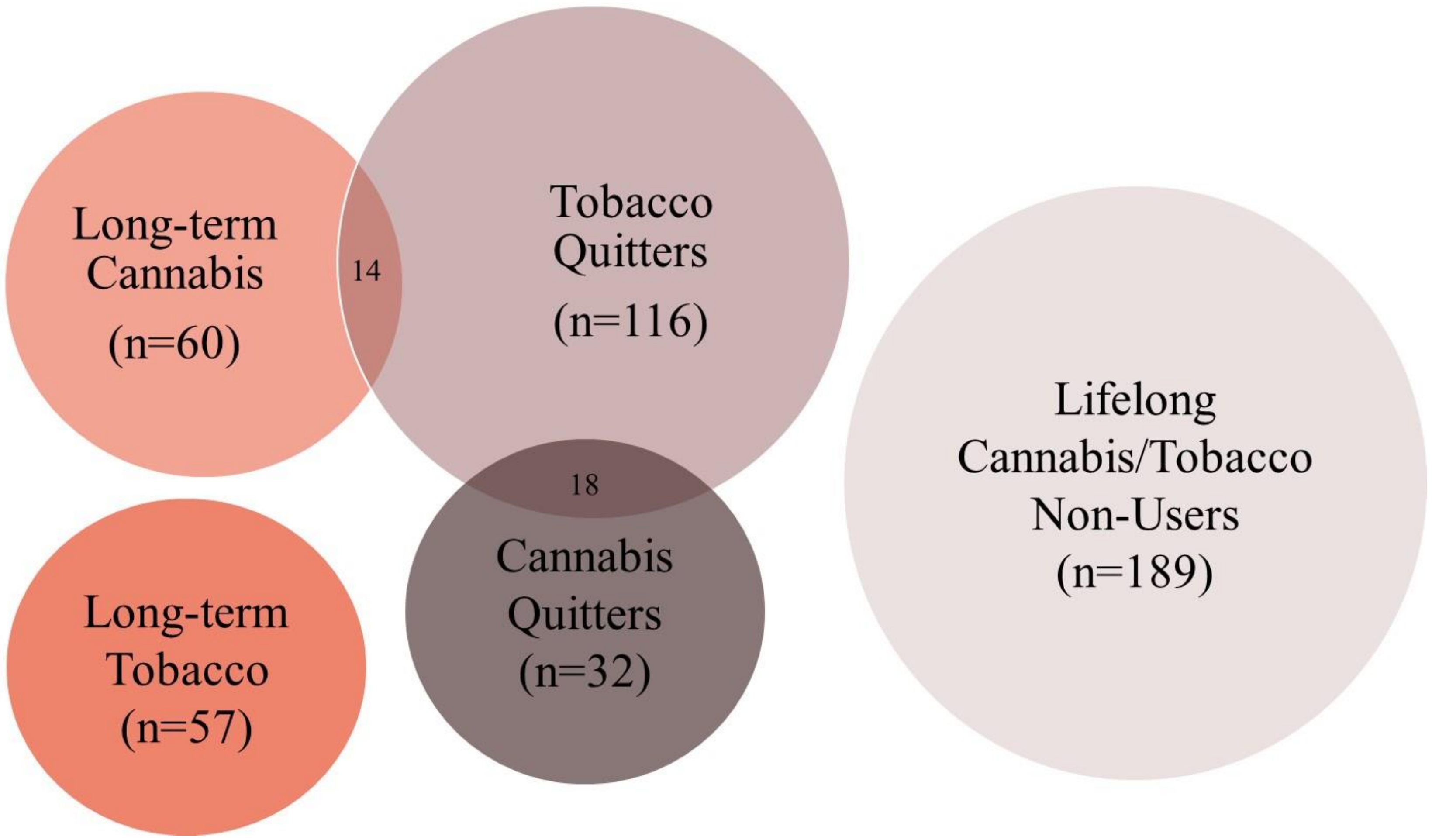

Figure S3. Circos plots of significant negative correlations between cannabis-related DNA methylation markers and gene expression probesets. Negative correlations reflect the expected direction of the association between DNA methylation and gene expression levels if direct regulation of gene expression through DNA methylation were to exist. Arrows originate from the genomic location of genes significantly correlated with DNA methylation probe values; the arrowhead is the location of the methylation probe under test. Correlations were calculated using Spearman's rho and Bonferroni-corrected p-value cutoffs for significance ($p = 1.02x10^{-6}$). Many of the genes correlated with DNA methylation have been previously implicated in smoking and smoking-related behavior (e.g. *LRRN3, AHHR, GPR15*).

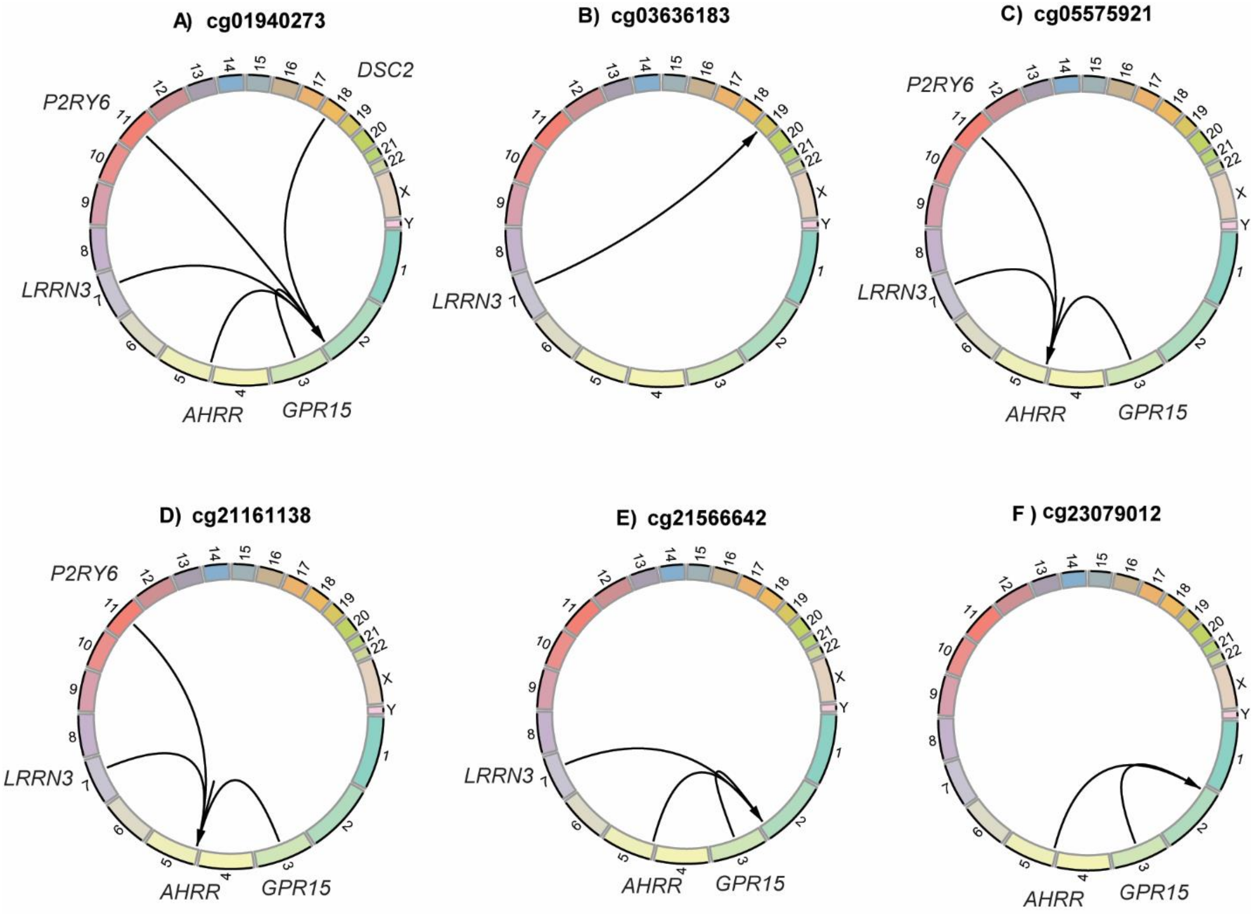

77. American Psychiatric Association. Diagnostic and Statistical Manual of Mental Disorders, Fourth Edition. Washington, DC: American Psychiatric Association; 1994.

78. American Psychiatric Association. Diagnostic and statistical manual of mental disorders. Third Edition, Revised. Washington, DC: American Psychiatric Association; 1987.

79. Elley WB, Irving JC. Revised socio-economic index for New Zealand. *New Zealand Journal of Educational Studies* 1976; 11(1):25-36.

80. Milne BJ, Caspi A, Crump R, Poulton R, Rutter M, Sears MR, et al. The validity of the family history screen for assessing family history of mental disorders. *Am J Med Genet B Neuropsychiatr Genet* 2009; 150B(1):41-9.

81. Dugué P-A, Jung C-H, Joo JE, Wang X, Wong EM, Makalic E, et al. Smoking and blood DNA methylation: an epigenome-wide association study and assessment of reversibility. *Epigenetics* 2020; 15(4):358-68.

82. Bermingham ML, Walker RM, Marioni RE, Morris SW, Rawlik K, Zeng Y, et al. Identification of novel differentially methylated sites with potential as clinical predictors of impaired respiratory function and COPD. *EBioMedicine* 2019; 43:576-86.

83. Zhang Y, Florath I, Saum K-U, Brenner H. Self-reported smoking, serum cotinine, and blood DNA methylation. *Environ Res* 2016; 146:395-403.

84. Joubert BR, Felix JF, Yousefi P, Bakulski KM, Just AC, Breton C, et al. DNA methylation in newborns and maternal smoking in pregnancy: genome-wide consortium meta-analysis. *Am J Hum Genet* 2016; 98(4):680-96.

85. Logue MW, Miller MW, Wolf EJ, Huber BR, Morrison FG, Zhou Z, et al. An epigenome-wide association study of posttraumatic stress disorder in US veterans implicates several new DNA methylation loci. *Clinical Epigenetics* 2020; 12:1-14.

86. Liu C, Marioni RE, Hedman ÅK, Pfeiffer L, Tsai P-C, Reynolds LM, et al. A DNA methylation biomarker of alcohol consumption. *Mol Psychiatry* 2018; 23(2):422-33.

87. Imboden M, Wielscher M, Rezwan FI, Amaral AF, Schaffner E, Jeong A, et al. Epigenome-wide association study of lung function level and its change. *Eur Respir J* 2019; 54(1).

88. Kashima K, Kawai T, Nishimura R, Shiwa Y, Urayama KY, Kamura H, et al. Identification of epigenetic memory candidates associated with gestational age at birth through analysis of methylome and transcriptional data. *Sci Rep-Uk* 2021; 11(1):3381.

89. Smith AK, Ratanatharathorn A, Maihofer AX, Naviaux RK, Aiello AE, Amstadter AB, et al. Epigenome-wide meta-analysis of PTSD across 10 military and civilian cohorts identifies methylation changes in AHRR. *Nat Commun* 2020; 11(1):5965.

90. Richmond RC, Sillero-Rejon C, Khouja JN, Prince C, Board A, Sharp G, et al. Investigating the DNA methylation profile of e-cigarette use. *Clin Epigenetics* 2021; 13:1-13.

91. Marioni RE, McRae AF, Bressler J, Colicino E, Hannon E, Li S, et al. Meta-analysis of epigenome-wide association studies of cognitive abilities. *Mol Psychiatry* 2018; 23(11):2133-44.

92. Kim KW, Park S-C, Cho H-J, Jang H, Park J, Shim HS, et al. Integrated genetic and epigenetic analyses uncover MSI2 association with allergic inflammation. *Journal of Allergy and Clinical Immunology* 2021; 147(4):1453-63.

93. Spiers H, Hannon E, Schalkwyk LC, Smith R, Wong CC, O'Donovan MC, et al. Methylomic trajectories across human fetal brain development. *Genome Res* 2015; 25(3):338-52.

94. Zhao N, Ruan M, Koestler DC, Lu J, Marsit CJ, Kelsey KT, et al. Epigenome-wide scan identifies differentially methylated regions for lung cancer using pre-diagnostic peripheral blood. *Epigenetics* 2022; 17(4):460-72.

95. Battram T, Yousefi P, Crawford G, Prince C, Babaei MS, Sharp G, et al. The EWAS Catalog: a database of epigenome-wide association studies. *Wellcome Open Research* 2022; 7:41.
